# The needs of healthcare personnel who provide home-based pediatric palliative care: a mixed method systematic review

**DOI:** 10.1186/s12913-023-10495-7

**Published:** 2024-01-09

**Authors:** Judith Schröder, Kirsti Riiser, Heidi Holmen

**Affiliations:** 1 Faculty of Health Sciences, Department of Nursing and Health Promotion, Oslo Metropolitan University, P.O. Box 4, Oslo, NO-0130 Norway; 2 Faculty of Health Sciences, Department of Rehabilitation Science and Health Technology, Oslo Metropolitan University, Oslo, Norway; 3 Division of Technology and Innovation, Intervention Centre, Oslo University Hospital, Oslo, Norway

**Keywords:** Pediatric palliative care, Home-based, Pediatric, Children, Healthcare personnel, Needs, Experiences

## Abstract

**Background:**

Families with children who have life-limiting or life-threatening illnesses often prefer to receive care at home to maintain a sense of normalcy. However, caring for children at home is different from caring for them in a hospital, and we do not know enough about the needs of healthcare personnel who provide home-based pediatric palliative care.

**Aim:**

The aim of this review was to systematically summarize, appraise and synthesize available quantitative, qualitative, and mixed methods research to identify the needs of healthcare personnel in home-based pediatric palliative care.

**Methods:**

We used the Joanna Briggs Institute methodology for mixed method systematic reviews and searched systematically in Medline, Embase, PsycINFO, CINAHL, Web of Science, AMED, and the Cochrane Library. Quantitative, qualitative and mixed methods studies from 2012 to 2021 reporting on healthcare personnel’s needs, experiences, perspectives, coping strategies, and/or challenges related to home-based pediatric palliative care were eligible for inclusion. The screening was conducted independently in pairs. The quantitative data were transformed into qualitative data and analyzed using thematic synthesis.

**Results:**

Overall, 9285 citations were identified, and 21 studies were eligible for review. Most of the studies were qualitative and interview-based. Few studies included healthcare personnel other than doctors and nurses. Three analytical themes were developed: (1) *being connected and engaged with the child and family*, (2) *being part of a dedicated team*, and (3) *ensuring the quality of home-based pediatric palliative care services.* Healthcare personnel strived to deliver high-quality, home-based pediatric palliative care. Establishing a relationship with the child and their parents, collaborating within a committed team, and having sufficient resources were identified as important needs influencing healthcare personnel when providing home-based pediatric palliative care.

**Conclusion:**

The findings underscore the importance of building trusting relationships among healthcare personnel, children, and families. It also emphasizes the significance of interdisciplinary collaboration that is effective, along with the presence of enough skilled personnel to ensure high-quality home-based pediatric palliative care. Further research is necessary to include healthcare personnel beyond doctors and nurses, as palliative care requires a team of professionals from various disciplines. Addressing the needs of healthcare personnel can ensure safe and professional palliative care for children at home.

**Supplementary Information:**

The online version contains supplementary material available at 10.1186/s12913-023-10495-7.

## Background

Palliative care for children with life-limiting or life-threatening diseases aims to ease suffering and enhance the quality of life of these children and their families [1]. Home-based services, which are often more common than inpatient services, especially in developed countries play a significant role in pediatric palliative care [2]. These services can allow the families to maintain a sense of normalcy in their homes, which is often preferred when caring for children with severe illness [3–5]. Healthcare personnel can provide specialized and professional care in a home environment, which can positively impact a family’s care experience and reduce costs [1, 3–6]. International standards in pediatric palliative care recommend that healthcare personnel are specialized in pediatric palliative care [6], but research indicates that there are vast variations in the competencies, content, and quality of services between countries and between urban and rural regions [4, 5]. In home-based pediatric palliative care, healthcare personnel experience more professional isolation because they predominantly work alone with limited professional collaboration and medical equipment [4, 5, 7, 8]. Furthermore, the emotional impact of providing pediatric palliative care is burdensome as healthcare personnel are confronted with children with unnaturally shortened lifespans [5, 7]. Palliative care services are often provided over a long period, which allows healthcare personnel to establish close relationships with the affected families, often leading to a strong emotional impact and making it challenging to maintain professional boundaries [5, 7]. Previous research on home-based services indicate that healthcare personnel struggle with organizational deficits, such as a lack of staff and equipment, which can be exhausting [9]. These organizational deficits can cause emotional reactions in healthcare personnel, such as anxiety and frustration and the feeling of not being able to deliver adequate pediatric palliative care [5, 9]. In addition to resources, it is important that organizations facilitate systematic and regular training in pediatric palliative care to promote the quality of home-based pediatric palliative care [4].

Home-based pediatric palliative care is a complex and challenging practice and few reviews have assessed and synthesized the existing research on this topic from different perspectives. Previous reviews have examined various aspects of home-based pediatric palliative care, such as community-based care in the U.S. [4], end-of-life care for children at home [5], or work-related stress among registered nurses [7]. However, none of these reviews have focused in identifying healthcare personnel`s needs in this setting. The aim of our mixed method systematic review is to identify the specific needs of healthcare personnel when providing home-based pediatric palliative care. We consider that understanding these needs of healthcare personnel is an appropriate approach to explore their roles and responsibilities, ultimately contributing to the improvement of practices in this field. Mixed method systematic reviews are widely recognized as the most robust method for addressing clinical questions and generating evidence that directly informs best practice [10]. To achieve a broad understanding of healthcare personnel`s needs, when they provided home-based pediatric palliative care, we conducted a mixed methods systematic review, which involved summarizing, appraising, and synthesizing available research studies that employed quantitative, qualitative, and mixed methods approaches.

## Methods

In this mixed method systematic review, we applied a convergent integrated approach transforming quantitative data to text, following the guidance of the Joanna Briggs Institute [10], to enable the use of the same synthesis method for both qualitative and quantitative data. We selected a mixed method review as this has been recommended when attempting to understand complex phenomena [10]. Needs can be conceptualized through expressed and felt needs [11]. These categories of needs were considered appropriate to examine the characteristics of healthcare personnel experiences when providing home-based pediatric palliative care. The protocol was registered in PROSPERO (CRD42021292865) a priori.

### Inclusion criteria

We used the sample, phenomenon of interest, design, evaluation, and research type (SPIDER) framework [12] to ensure a comprehensive search and to determine explicitly whether a study was eligible for inclusion in the review (Table 1). This review centers on contemporary research regarding practices in home-based pediatric palliative care and encompasses studies published between 2012 and 2021, reflecting the evolution of pediatric palliative care research over the past decade [13]. The articles that were not explicit about whether palliative care was provided in a child’s home or if healthcare personnel provided palliative care to adults or children were excluded. The review only included articles published in English, German, or Scandinavian languages due to limited funding, time, and resources. Articles in other languages were excluded during the full-text screening to assess the potential impact of language exclusion on the review’s internal validity [14].

**Table 1:** Inclusion and exclusion criteria according to sample, phenomenon of interest, design, evaluation, research type (SPIDER) [12]

Category	Inclusion	Exclusion
Sample (S)	Studies reporting on samples of healthcare personnel who belong to medical, nursing, or other disciplines who are trained to provide healthcare services	Studies reporting on a sample of other professions involved in pediatric palliative care but not working in healthcare services Studies reporting on a mixed sample of participants, where the results relating to the healthcare personnel cannot be separated Studies reporting on a sample of healthcare personnel in an educational role/position
Phenomenon of interest (PI)	Home-based (delivered in a child’s home) pediatric (0–18 years) palliative care, regardless of the diagnosis	Studies reporting on pediatric palliative care in inpatient hospitals, nursing home hospices, free-standing hospices, or community-based intellectual disability services, or with a mixed sample, where the results cannot be separated to pediatric palliative care at home Studies reporting on a sample of children with diagnoses not requiring palliative care Studies reporting on samples of young adults (> 18 years) only
Design (D)	Studies published in peer-reviewed journals - Quantitative descriptive research - Incidence or prevalence study without comparison group - Survey - Grounded theory - Ethnography research - Case studies - Observational studies - Phenomenological research - Narrative research - Qualitative description - Mixed methods design	- Systematic reviews - Master and PhD theses - Conference abstracts or posters - Editorials - Comments - Protocols, - Non-empirical papers, or letters
Evaluation (E)	Studies reporting on healthcare personnel’s descriptions, reports, narratives, or opinions about their needs, experiences, perspectives, coping strategies, and/or challenges	Studies reporting on healthcare personnel`s descriptions, narratives, or opinions of children and/or their parents’ needs
Research type (R)	Qualitative, quantitative, or mixed methods research	Not applicable

### Search strategy and information sources

To find relevant evidence that explored the aim of our review, we collaborated with university librarians to identify relevant keywords and index terms to develop the search strategy. In October 2021, an initial test of the search strategy was conducted by the university librarians using the Medline database. This enabled us to evaluate the relevance of the retrieved literature and refine our strategy with additional keywords and index terms. The purpose of this preliminary test search was to develop a systematic and comprehensive search strategy. Based on this testing process, the university librarians performed the final systematic search in seven databases, namely, Medline, Embase, PsycInfo, Cumulative Index to Nursing and Allied Health Literature (CINAHL), Web of Science, Allied and Complementary Medicine (AMED), and the Cochrane Library, on December 9, 2021. An updated search was conducted by the librarian on December 5, 2023, utilizing the same search strategy and databases as before.

In each database, the keywords, synonyms, and subject headings to palliative care (1/ Phenomenon of interest), child/adolescent (2/ Phenomenon of interest), homecare services (3/ Phenomenon of interest), and healthcare personnel (4/ Sample) were used to compose the search strategies. The complete search strategies are available in Additional file 1. The phenomenon of interest was divided into four separate terms, which were combined (AND).

### Study selection

All the identified citations were uploaded to Covidence [15]. Covidence identifies duplicate citations by checking the title, year, and volume. However, any remaining duplicates were identified manually by the first author. Pairs of reviewers independently screened the titles, abstracts, and full text based on the preestablished eligibility criteria. JS screened all the studies, whereas HH and KR split the screening of the titles, abstracts, and full text, and each assessed one-half. The citations were discussed and disagreements regarding abstract or full text were resolved by consensus between all three authors.

### Assessment of methodological quality

The methodological quality of the included studies was assessed independently in Covidence by JS and HH using the mixed methods appraisal tool (MMAT) version 2018 [16]. MMAT is a critical appraisal tool for five different study designs in qualitative, quantitative, and mixed methods studies [16]. In this review, MMAT was used to independently assess qualitative research (MMAT_1_), quantitative descriptive studies (MMAT_4_), and mixed methods studies (MMAT_5_). Questions about the quality domains of the chosen study design were answered with “Yes,” “No,” and “Can’t tell.” All the quality domains were considered important, and no studies were excluded from the analysis based on their methodological quality.

### Data extraction and transformation

The characteristics of the included studies were extracted in Covidence using a tailored data collection form (Additional file 2), which was inspired by the SPIDER framework [12], and developed a priori. JS extracted specific details about the author(s), year, country, sample, phenomena of interest, research design, evaluation, and results. HH checked all the collected data. JS reviewed the full texts of all the studies included, and all the authors agreed to extract the text, tables, and data of all the figures labeled as “results” or “findings” for the thematic synthesis. Both quantitative and qualitative data can address our research question and can be combined once transformed into the same format [10]. We transformed quantitative data from included studies into textual descriptions, enabling their integration with qualitative data through a process known as “qualitizing” [10]. In our review this approach involved narrative interpretation of quantitative results, which primarily consisted of descriptive statistics like mean values or percentages. Additional file 4 provides examples of transformed quantitative data into qualitative data. JS extracted all the text and imported it into NVivo 12.

### Data synthesis and integration

To ensure that our research question guided the synthesis, we explicitly focused on data from the included studies that told something about expressed and felt needs in relation to the scope, responsibilities, and tasks of healthcare personnel in home-based pediatric palliative care [11]. JS and HH independently read the document with the result sections of all the included studies to determine their relevance in relation to the research question. As described by Thomas and Harden [17], in the first stage of the thematic synthesis, JS coded each line of the extracted data according to its meaning and content. To identify relevant results describing the needs of healthcare personnel, the data were carefully examined, and results that could be coded as needs were included. Examples of coded results are presented in additional file 4. This process generated 79 codes, which were closely linked to the content and meaning of the text. In stage two, JS read and reread all 79 codes to summarize the codes with similar meanings and developed descriptive themes that were close to the content. These descriptive themes were presented to HH to facilitate a discussion on the understanding and interpretation of the themes and to consider the meaning of all the findings. In stage three, the descriptive themes were interpreted and discussed by all authors in the context of the review question. Additional file 5 presents examples of all stages of our thematic synthesis.

## Results

### Characteristics of the included studies

**Fig. 1:**
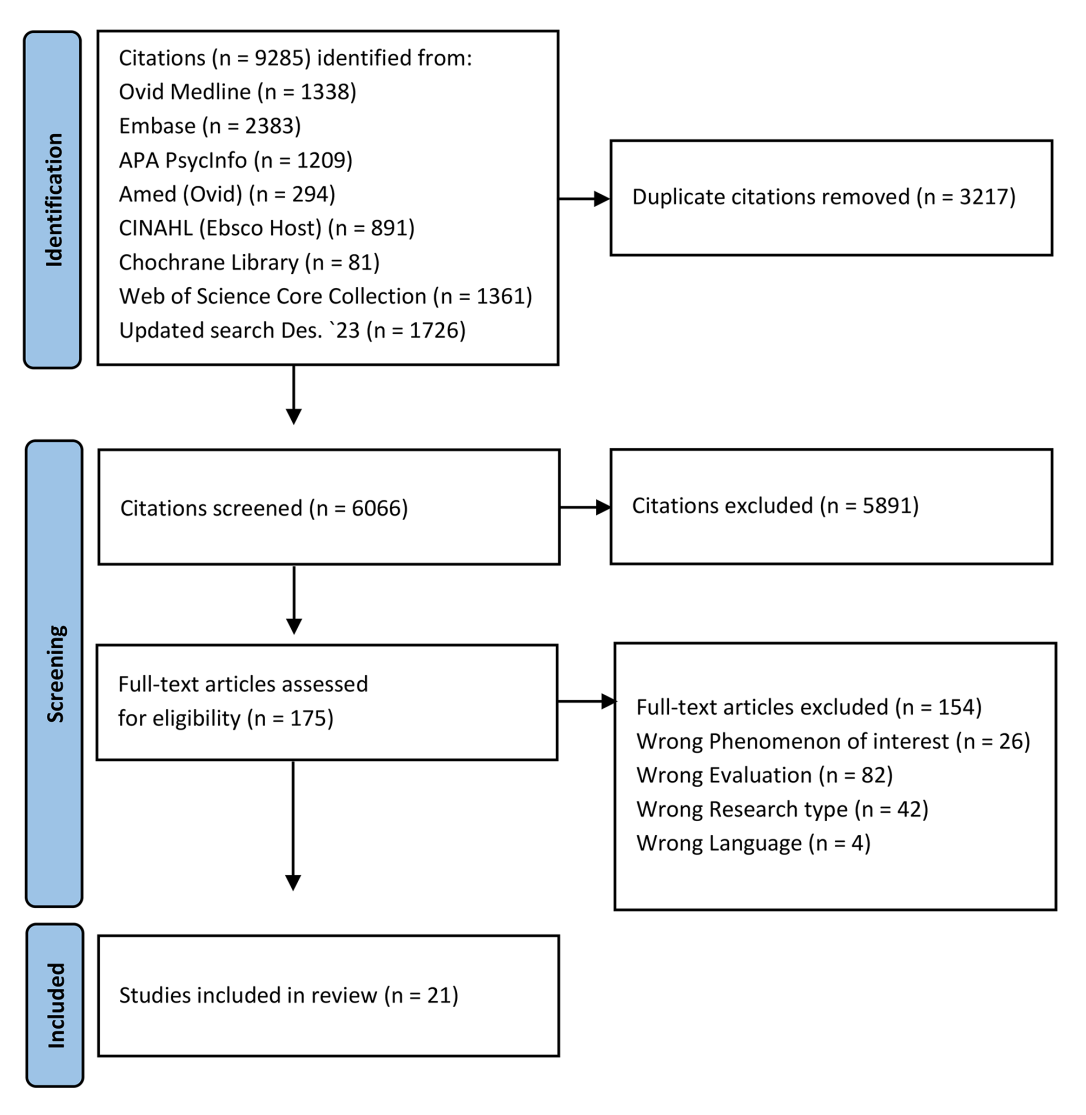
PRISMA flow diagram [39] of the citations that were screened and included in the systematic review

The search identified 9285 citations. After deduplication, the titles, and abstracts of 6066 unique citations were screened for eligibility according to the inclusion criteria. Altogether, the full text of 175 citations were evaluated, which resulted in 21 studies [18–38] that were eligible for review (Fig. 1).

The included studies originated from Europe (n = 16) [18, 20, 22, 24–26, 28–33, 35–38], North America (n = 2) [27, 34], Australia (n = 1) [19], Africa (n = 1) [21], and Asia (n = 1) [23]. Thirteen studies were qualitative and took the form of interview studies [19–23, 26–29, 34, 35, 37, 38]. Seven studies were quantitative and presented survey data [18, 24, 30–33, 36], and study had a mixed methods approach [25]. Physicians (n = 333), including pediatricians [22, 24, 25, 30, 35, 37, 38], were the largest participant group, followed by nurses (n = 219) [18, 19, 21–23, 27–29, 34–38]. Some studies involved other healthcare personnel, such as counsellors, assistant nurses, occupational therapists, physiotherapists, dietitians [22, 35, 37], paramedics, psychosocial professionals, and other professionals [31, 35, 37, 38], and home-based care workers [21]. Two studies presented participants as external stakeholders, in-patient hospital staff, and hospice at home teams [20] or as allied health clinicians [19]. Two studies did not report the exact sample size [26, 32].

### Methodological quality

The appraisal of the included studies’ methodological quality is presented in full in Additional file 3. The majority of the included qualitative studies clearly described the collection of the data and analyses and provided representative quotations to justify the findings. In one of the studies [20], the findings were less obvious because of insufficient quotes, while another study [21] had insufficient details to determine the method of data collection. These two studies could therefore not be evaluated in relation to coherence between the data sources and interpretation.

The quality of the quantitative studies included in this review varied, and many lacked transparency. The categorial differences between the respondents and nonrespondents were only possible to evaluate in one of the studies [30]. In three studies [30–32], the variables were defined, and the measurements were appropriated to answer the research question. Four studies [18, 24, 30, 32] reported a description of the target population and sample and the inclusion and exclusion criteria. None of the studies used validated questionnaires.

One mixed methods study [25] was included and reviewed. However, the main focus of the study was on the quantitative findings, and the qualitative elements were not described in detail. As a result, it was challenging to evaluate the methodological quality of the qualitative aspects of the study.

### Overview of themes

**Fig. 2:**
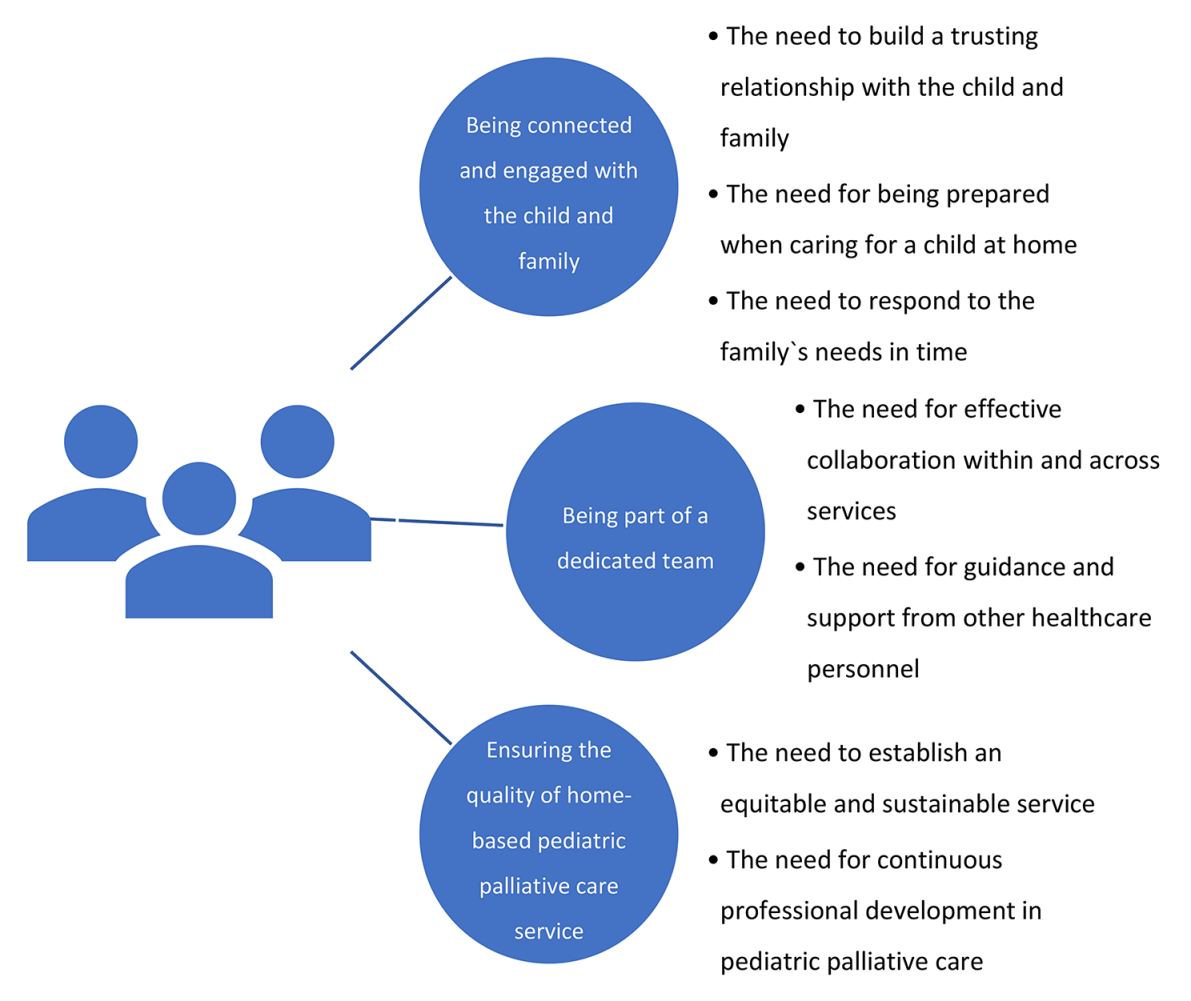
Overview of the analytical and descriptive themes

Our thematic analysis provides a summary and synthesis of the needs of healthcare personnel. Three analytical themes were developed: (1) *being connected and engaged with the child and family*, (2) *being part of a dedicated team*, and (3) *ensuring the quality of home-based pediatric palliative care* (Fig. 2). The themes and corresponding descriptive subthemes are presented below.

### Theme 1: being connected and engaged with the child and family

The healthcare personnel described the children’s homes as unfamiliar places but acknowledged that families preferred to receive care at home. Sometimes healthcare personnel did not know the child before the first home visit [29]. To ensure that the care they provided met the needs of the child and family at home and to make decision-making a shared responsibility between healthcare personnel and parents, healthcare personnel stressed **the need to build a trusting relationship with the child and their family** [18, 19, 22, 23, 25, 26, 28, 29, 34, 35, 37, 38]. Healthcare personnel found that establishing a trusting relationship made families feel safe and protected and had the potential to alleviate the burdens experienced as their children’s illnesses progressed [22, 23, 25, 26, 35, 37]. They also reported that gaining the trust of a child can be challenging [22, 23], but trust is needed to ensure open discussions and truth-telling and to involve the child in their care [21, 22, 28]. Healthcare personnel expressed that knowing and understanding the family helps clarify the responsibilities between the healthcare personnel and the family [27, 35] and makes it easier to assess if the family is coping at home [22, 23]. Telehealth can be useful in facilitating this relationship if large geographical distances are involved, but this was experienced by healthcare personnel as a less personal communication form compared to physical home visits [19, 34].

According to the perspective of healthcare personnel, the family’s perception of palliative care solely as end-of-life care may pose a challenge in establishing a trusting relationship early in a child’s disease [30, 36, 37]. Late referrals from inpatient to outpatient services constituted another barrier for healthcare personnel when trying to establish a trusting relationship with a child and their family [22–24, 29]. Maintaining professional boundaries was mentioned as an important aspect of a trusting relationship, but balancing familiarity and emotional involvement with a level of detachment could be challenging [18, 22, 25, 26, 28, 29, 35, 37]. Healthcare personnel who provide home-based pediatric palliative care needed to be aware of the emotional impact of their work on themselves [35].

Home-based pediatric palliative care was described as a type of health care that was provided in the individual home where the child and family lived. According to healthcare personnel, this made home care an unpredictable environment, with limited resources and the need to adapt to different families [37]. Therefor healthcare personnel emphasized the importance of standing out as skilled professionals when working alone in a family’s home [20, 22, 23, 25–27, 29, 37]. Appearing less knowledgeable or unready was found to cause distress [18, 20, 22–27, 29–31, 33, 36]. This distress emphasized **the need for being prepared when caring for a child at home**. A need for being prepared was particularly evident among the healthcare personnel who mainly provided palliative care for adults and were, therefore, less experienced with young patients [22, 23, 27]. This included being prepared to manage medical devices, assess symptoms, adjust medication dosages [24, 26, 30, 33], and handle ethical dilemmas related to life-prolonging interventions and alternative medicine [21, 28]. Additionally, healthcare personnel needed to adopt an attitude of accepting death as a natural occurrence rather than a professional failure; this shift had to happen for their emotional well-being [37]. Once home-based pediatric palliative care was established, healthcare personnel understood that care in the home favored a holistic approach to the needs of the children and their families [37, 38]. Age-appropriate communication with the children and open communication with families experiencing emotional stress were also identified as crucial factors [21, 23, 28, 29, 37].

To maintain a family’s trust and provide safe care, healthcare personnel **needed to respond to the family’s needs in time**. Home-based pediatric palliative care was perceived as more time-consuming and less predictable than home-based palliative care for adults [18, 22–24, 26]. According to healthcare personnel, their busy workloads made it difficult for them to manage children’s medical procedures and emergency calls from families and to have time to be present for children and families experiencing emotional stress [18, 22, 23]. On the other hand, providing home-based pediatric palliative care was deemed to offer a unique experience that justified allocating sufficient time, even outside of working hours [18, 22, 24, 25, 29].

### Theme 2. Being part of a dedicated team

The healthcare personnel in several studies reported that optimal home-based pediatric palliative care should involve different professionals from within and across healthcare services [19, 20, 22–24, 26, 31, 34, 37]. Services like psychosocial support, play therapists, respite services, and specialized pediatric palliative care services helped to ensure that families could fulfill their wish to care for their children at home. However, effective working relationships and communication across different healthcare services for joint patient care were experienced as challenging [22–24, 31, 33]. This was partly due to the rarity of palliative care for children in community services, which meant that personnel from different healthcare services often found themselves working together for the first and only time [26]. Accordingly, **the need for effective collaboration within and across services** was essential. For healthcare personnel effective collaboration between healthcare services was entailed as a clear understanding of each other’s roles and responsibilities and required functional routines for the exchange of information [19, 20, 22, 24, 26, 28, 30, 31, 33, 36–38]. In addition, healthcare personnel needed to change their mentality to be flexible, proactive, decisive, and willing to work as a team [37]. Healthcare personnel can become confused, frustrated, or even feel threatened when collaboration is poorly planned and managed [22, 24, 28–31]. Introductory meetings between specialized pediatric palliative care teams and the home care services could make healthcare personnel feel more prepared for home-based pediatric palliative care [36]. Three studies [19, 34, 36] examined the use of telehealth, like video conferences, in home-based pediatric palliative care, and it was reported to be an efficient method for maintaining working relationships and enhanced communication. Video conferences permitted personnel from different healthcare services to be brought up to date and included in discussions regardless of the geographical location of the services [19, 34]. Collaboration seemed to cause in-hospital healthcare personnel to hand more responsibility over to local healthcare personnel [19], and children could thus be transferred home earlier because the hospital staff knew the local healthcare personnel from different services well [22].

Simultaneously, close collaboration enhanced the transfer of knowledge and skills from specialized hospital personnel to homecare personnel with less competence in pediatric palliative care [18, 19, 22, 24, 25, 27, 30–32]. **The need for guidance and support from other healthcare personnel** were echoed in the included studies. The healthcare personnel who collaborated with pediatric palliative care teams considered these specialized teams to be the most useful resources when providing palliative care at the homes of children [18, 22, 24, 25, 27, 31, 32, 36]. Through that collaboration, the healthcare personnel received guidance and clarity regarding the symptoms to be expected, clinical assessments, and medication dosages and were able to discuss dilemmas. Notwithstanding, support from colleagues in the organization was also highlighted as important [23–25, 27, 29, 35]. When healthcare personnel worked alone with a child in their home and dealt with occasionally stressful work tasks, colleagues served as a source of emotional support. Reflective sharing, debriefing sessions, joint visits, or just having had the opportunity to reach out to a colleague by phone promoted coping and made healthcare personnel feel more supported at work [22–25, 27, 29, 30, 35].

### Theme 3. Ensuring the quality of home-based pediatric palliative care services

The findings of our review reflected that healthcare personnel had **the need to establish equitable and sustainable services **to ensure optimal care for children at home.

Home-based pediatric palliative care services seemed to consist of small local teams and pediatric patients spread over wide geographical areas [19, 22, 23, 29, 34]. The limited availability of appropriately skilled healthcare personnel was a barrier to provide flexible home-based pediatric palliative care, and especially out-of-hours care [18, 20, 26, 28, 38]. Small teams did not provide healthcare personnel with opportunities to rest and relinquish their practical and emotional responsibilities [26, 29]. Healthcare personnel requested clear guidelines and protocols to ensure that all involved personnel provide equal care to children in need of home-based pediatric palliative care as well as their families [20, 23, 28].

The scarcity of pediatric patients compared to adults and the fundamental differences between caring for children versus adults were highlighted as reasons for healthcare personnel’s lack of knowledge and skills in pediatric palliative care [20, 22–29, 32, 33]. Healthcare personnel, therefore, expressed a **need for continuous professional development in pediatric palliative care**. As healthcare personnel shared their experiences, they emphasized the importance of the workplace having supported them with time, resources, and access to relevant courses and in-service training. This included not only theoretical courses but also practical guidance and hands-on learning, all tailored to their specific needs [20, 22, 24, 27, 37]. The topics specifically mentioned for training were clinical assessments, communication, and strategies for self-care (Table 2) [18–20, 22–27, 29–31, 33, 37, 38].

**Table 2:** Specific topics for training and experience in pediatric palliative care

Knowledge and skills in clinical assessments	• Pediatric pathophysiology and disease progression • Pharmacologic and nonpharmacologic symptom management • Medication doses • Medical devices and equipment
Knowledge and skills in communication	• Age or developmentally appropriate interaction with children • Navigating difficult conversations with children and their families • Responding to families’ emotional reactions appropriately • Cultural/contextual sensitivity
Knowledge and skills for self-care strategies	• Balancing and coping with emotions by being mentally focused and emotionally prepared • Formal and informal debriefings with colleagues • Establishing boundaries

The emotional toll of providing home-based pediatric palliative care became manageable for healthcare personnel when time is dedicated to debriefing, or routines for supervision were established [22, 23, 32, 35]. Healthcare personnel recounted that workplace managers would demonstrate their appreciation for professional development by facilitating and funding courses and educational opportunities [22, 27].

## Discussion

This systematic review presents a summary of studies and a synthesis of healthcare personnel’s needs when providing home-based pediatric palliative care. Most of the studies investigated the needs of doctors and nurses more than those of other healthcare personnel. The methodological quality of the included primary studies was varying. The difficulties with feasible and rigorous quantitative research are a well-known phenomenon in pediatric palliative care research [40]. The small population, heterogeneity of the illnesses, heterogeneity among providers, low response rates, and ethical concerns are some of the challenges. Despite these issues, previous research can serve as a foundation for future systematic research [6, 40, 41].

The reviewed studies illustrated that home-based pediatric palliative care can be provided by various services, from hospital- to community-based services. These different models of care demonstrate that home-based pediatric palliative care depends on the country and region, local regulations, and access to resources [5, 42]. To better inform future service development in home-based pediatric palliative care, researchers should provide more details on characteristics of the home-based service and how it is delivered in practice [5, 7, 42, 43].

The need to establish trusting relationships was highlighted as important but challenging when providing home-based pediatric palliative care. This aligns with previous research, which has noted that trust is an important prerequisite in care [4, 5, 9, 43]. A trusting relationship also contributes to the assessment of the individual needs of children and their families and gives healthcare personnel the opportunity to respond proactively [4, 5, 43, 44]. Previous research in home-based care has shown that healthcare personnel adapt from being a person outside of the family to becoming part of the family [4, 5, 7, 43]. A recent systematic integrative review described nurses’ competences in collaborating with patients and families and how patients value nurses’ personal involvement [43]. Findings indicates that being personally involved is not contradictory to being professional, especially when providing care in a patient’s home [43]. Healthcare personnel who become a part of the family and show personal involvement can help normalize the family’s situation when they rely on professional help for their child. However, on the other hand research shows that a close relationship between healthcare personnel and the family can blur professional boundaries, leading to work-related stress [5, 7]. Caring for children with palliative needs can also impact healthcare personnel emotionally, potentially making them feel unprofessional [7, 9, 45]. Implementing mental health support interventions, such as training and supervision, is of paramount importance in the workplace. These interventions not only encourage reflective clinical practice and professional progression, but they also help alleviate the stress that healthcare personnel frequently face in challenging work situations over long periods, such as those encountered in pediatric palliative care [7, 9, 45].

Standards for pediatric palliative care describe care as an interdisciplinary and complex service, and the reviewed studies showed that working in a team affected the needs of healthcare personnel [6]. A lack of collaboration across healthcare services can be a source of stress [7], and this was echoed throughout the studies included in our review. A recent systematic integrative review that investigated the distinct levels of palliative care provision (palliative approach, generalized palliative care, and specialized palliative care) and the associated competence showed how the provision of different levels of palliative care is not sufficiently defined [43]. Clarifying roles and responsibilities can help ensure that all involved healthcare personnel take responsibility for and contribute to achieving common care goals [4, 41, 44]. Coordinated collaboration also contributes by providing a source of guidance from more experienced colleagues to healthcare personnel who are less experienced in providing pediatric palliative care [4, 41, 46]. The advantage of interprofessional collaboration was also discussed in the included studies. Understanding and appreciating each other’s roles between different providers can secure continuity of care [4, 5, 7, 41, 44].

Establishing a service with competent professionals and overarching collaboration to provide home-based care for children and their families was identified as difficult in most of the studies included in this review. As confirmed in previous research, staff shortages are a key stressor for healthcare personnel [5, 7]. In addition, workplaces must enable healthcare personnel to acquire basic levels of knowledge and skills in pediatric palliative care via education, affiliations with professional organizations, and networking opportunities [4, 7, 46]. However, the included studies illustrated that healthcare personnel struggle to acquire sufficient and relevant palliative care competencies because the number of children who would benefit from palliative care is low, and the opportunities for education and training are thus limited. Mentoring from colleagues was highlighted as an effective measure that requires few resources, as mentoring can take place in the interdisciplinary groups established around children [4, 7]. Digital collaboration can provide more flexible care for children and their families, but also to a greater extent, cover the needs of healthcare personnel in terms of collaboration, coordination, and competence enhancement [47–49]. Only two of the reviewed studies investigated digital solutions in home-based pediatric palliative care.

### Limitations

Although we strived to conduct our review rigorously according to the guidelines of the Joanna Briggs Institute, it has some limitations. The use of various terms to describe home-based pediatric palliative care based on different services in different countries may have resulted in missed studies that were not indexed with the keywords, synonyms, or subject headings used in our search strategy. To reduce the risk of missing out eligible studies, we accepted a high number of records to review; consequently, leading to the exclusion of a high number of records at the title and abstract screening. These records mainly did not agree with our inclusion criteria, such as phenomenon of interest, or the records were not reporting research. The inclusion process may have been affected by the individual reviewers’ interpretations of “needs”. While there have been studies exploring different aspects of home-based pediatric palliative care, these studies did not explicitly aim to identify the needs of healthcare personnel. As reviewers, we had to interpret the findings of these studies to determine what they reveal about the needs of healthcare personnel in this context. Finally, many of the studies were from high-income countries, which made it difficult to generalize the results to low- and middle-income countries.

## Conclusions

Healthcare personnel have various needs that must be met for them to provide professional care for children and their families at home. These needs include being connected with the child and their family, being part of a dedicated team, and ensuring service quality. This review confirmed that providing home-based palliative care services varies from country to country. We found few studies on healthcare personnel other than doctors and nurses, even though palliative care is intended to be an interdisciplinary service. Trusting relationships between children, families, and healthcare personnel are fundamental when providing palliative care for children at home. Close relationships give healthcare personnel the opportunity to identify and meet families’ needs and provide proactive, personalized care. Coordinated interdisciplinary collaboration between healthcare personnel within and across services contributes to the provision of holistic care for families as well as emotional and professional support. Sufficient competent healthcare personnel and adequate training and guidance are essential for providing home-based pediatric palliative care. Healthcare services should ensure that their personnel are well-trained and supported to provide high-quality care in the home setting. Further research is required to determine how healthcare services can be organized to meet the needs of the healthcare personnel who provide palliative care for children in their homes.

### Electronic supplementary material

Below is the link to the electronic supplementary material.

Supplementary Material 1

Supplementary Material 2

Supplementary Material 3

Supplementary Material 4

Supplementary Material 5

## Data Availability

All the data generated and analyzed during this study have been included in this published article in line with the search strategy presented in the PRISMA flow diagram in Fig. 1. Characteristics of the included articles on which this review was based are attached as Additional file 2.

## References

[1] Connor SR, editor. Global Atlas of Palliative Care 2nd Edition. London, UK: Worldwide Palliative Care Alliance; 2020. Available from: http://www.thewhpca.org/resources/global-atlas-on-end-of-life-care.

[2] Arias-Casais N, Garralda E, Pons JJ, Marston J, Chambers L, Downing J (2020). Mapping Pediatric Palliative Care Development in the WHO-European Region: children living in Low-to-Middle-Income Countries are less likely to Access it. J Pain Symptom Manage.

[3] Winger A, Kvarme LG, Løyland B, Kristiansen C, Helseth S, Ravn IH (2020). Family experiences with palliative care for children at home: a systematic literature review. BMC Palliat Care.

[4] Boyden JY, Curley MAQ, Deatrick JA, Ersek M (2018). Factors Associated with the Use of U.S. community–based Palliative Care for Children with Life-limiting or life-threatening illnesses and their families: an integrative review. J Pain Symptom Manage.

[5] Malcolm C, Knighting K, Taylor C (2020). Home-based end of Life Care for Children and their families – A systematic scoping review and narrative synthesis. J Pediatr Nurs.

[6] Benini F, Papadatou D, Bernadá M, Craig F, De Zen L, Downing J (2022). International standards for Pediatric Palliative Care: from IMPaCCT to GO-PPaCS. J Pain Symptom Manage.

[7] Lee J, Clarke S, Lynn F (2021). Understanding the causes of Work-related stress among registered nurses working with children at home: an integrative literature review. Compr Child Adolesc Nurs.

[8] Wager J, Kubek LA, Brenner M, Calmanti S, Doyle C, Lövgren M (2022). Expert survey on coverage and characteristics of pediatric palliative care in Europe – a focus on home care. BMC Palliat Care.

[9] Rabbetts L, Harrington A, Breaden K (2020). Nurses’ experience of providing home-based palliative care in the country setting: an integrated literature review. Int J Nurs Pract.

[10] Lizarondo L, Stern C, Carrier J, Godfrey C, Rieger K, Salmond S et al. Chapter 8: Mixed methods systematic reviews. JBI, 2020 [cited 2023 Apr 23]. In: JBI Manual for Evidence Synthesis [Internet]. [cited 2023 Apr 23]. Available from: https://synthesismanual.jbi.global/.

[11] Royse DD, Staton-Tindal M, Badger K, Webster MJ, What Is Needs. Assessment? 2009. In: Needs assessment [Internet]. New York: Oxford University PressPocket guides to social work research methods; [3–20].

[12] Cooke A, Smith D, Booth A, Beyond PICO (2012). The SPIDER Tool for qualitative evidence synthesis. Qual Health Res.

[13] Sisk BA, Feudtner C, Bluebond-Langner M, Sourkes B, Hinds PS, Wolfe J. Response to suffering of the seriously Ill child: a history of Palliative Care for Children. Pediatrics. 2020;145(1). 10.1542/peds.2019-1741 PMC6939842 31806669

[14] Neimann Rasmussen L, Montgomery P (2018). The prevalence of and factors associated with inclusion of non-english language studies in Campbell systematic reviews: a survey and meta-epidemiological study. Syst Rev.

[15] Veritas Health Innovation (2022). Covidence systematic review software.

[16] Hong QN, Fàbregues S, Bartlett G, Boardman F, Cargo M, Dagenais P (2018). The mixed methods Appraisal Tool (MMAT) version 2018 for information professionals and researchers. Educ Inf.

[17] Thomas J, Harden A (2008). Methods for the thematic synthesis of qualitative research in systematic reviews. BMC Med Res Methodol.

[18] Bertrand A, Veyet V, Goy F, Cervos M, Schell M. Pediatric palliative care at home by Home Care Unit: how home nurses feel? Supportive Care in Cancer. 2021. 10.1007/s00520-021-06623-w 34661749

[19] Bradford NK, Young J, Armfield NR, Herbert A, Smith AC (2014). Home telehealth and paediatric palliative care: clinician perceptions of what is stopping us?. BMC Palliat Care.

[20] Brenner M, Connolly M, Cawley D, Howlin F, Berry J, Quinn C (2016). Family and healthcare professionals’ perceptions of a pilot hospice at home programme for children: a qualitative study. BMC Palliat Care.

[21] Campbell LM, Amin N (2013). Dilemmas of telling bad news: paediatric palliative care providers’ experiences in rural KwaZulu-Natal, South Africa. SAJCH South African Journal of Child Health.

[22] Castor C, Hallstrom I, Hansson H, Landgren K (2017). Home care services for sick children: Healthcare professionals’ conceptions of challenges and facilitators. J Clin Nurs.

[23] Chong L, Abdullah A (2017). Community Palliative Care nurses’ challenges and coping strategies on delivering home-based Pediatric Palliative Care. Am J Hospice Palliat Med.

[24] Kremeike K, Eulitz N, Junger S, Sander A, Geraedts M, Reinhardt D (2012). Paediatric palliative home care in areas of Germany with low population density and long distances: a questionnaire survey with general paediatricians. BMC Res Notes.

[25] Neilson S, Gibson F, Jeffares S, Greenfield SM (2020). GPs and paediatric oncology palliative care: a Q methodological study. BMJ Supportive & Palliative care.

[26] Neilson SJ, Kai J, McArthur C, Greenfield S (2013). Using social worlds theory to explore influences on community nurses’ experiences of providing out of hours paediatric palliative care. J Res Nurs.

[27] Porter AS, Zalud K, Applegarth J, Woods C, Gattas M, Rutt E et al. Community Hospice Nurses’ Perspectives on Needs, Preferences, and Challenges Related to Caring for Children with Serious Illness. JAMA Netw Open. 2021;4(10) (no pagination). 10.1001/jamanetworkopen.2021.27457 PMC8491107 34605916

[28] Reid FC (2013). Grief and the experiences of nurses providing palliative care to children and young people at home. Nurs Child Young People.

[29] Reid FC (2013). Lived experiences of adult community nurses delivering palliative care to children and young people in rural areas. Int J Palliat Nurs.

[30] n der Geest IMM, Bindels PJE, Pluijm SMF, van der Michiels EMC, Pieters R (2017). Home-based Palliative Care for Children with Incurable Cancer: long-term perspectives of and impact on General practitioners. J Pain Symptom Manag.

[31] Verberne LM, Kars MC, Schepers SA, Schouten-Van Meeteren AYN, Grootenhuis MA, Van Delden JJM. Barriers and facilitators to the implementation of a paediatric palliative care team. BMC Palliat Care. 2018;17(1) (no pagination). 10.1186/s12904-018-0274-8 PMC5810030 29433576

[32] Vollenbroich R, Duroux A, Grasser M, Brandstatter M, Borasio GD, Fuhrer M (2012). Effectiveness of a pediatric palliative home care team as experienced by parents and health care professionals. J Palliat Med.

[33] Wallace E, Twomey M, O’Reilly M (2012). Challenges in the management of pediatric central venous access devices in the community. Pediatr Hematol Oncol.

[34] Weaver MS, Neumann ML, Navaneethan H, Robinson JE, Hinds PS (2020). Human touch via Touchscreen: rural nurses’ experiential perspectives on Telehealth Use in Pediatric Hospice Care. J Pain Symptom Manag.

[35] Rico-Mena P, Güeita-Rodríguez J, Martino-Alba R, Castel-Sánchez M, Palacios-Ceña D (2023). The emotional experience of caring for children in Pediatric Palliative Care: a qualitative study among a home-based Interdisciplinary Care Team. Child (Basel).

[36] Larsen SHK, Bording I, Bjergegaard M, Buchreitz J, Mouritzen BT, Brix L (2023). Paediatric end-of-life care at home. Int J Palliat Nurs.

[37] Rico-Mena P, Güeita-Rodríguez J, Martino-Alba R, Chocarro-Gonzalez L, Sanz-Esteban I, Palacios-Ceña D (2023). Understanding pediatric palliative care within interdisciplinary palliative programs: a qualitative study. BMC Palliat Care.

[38] Santana-Medina J, Rodríguez-Suárez CA (2023). Home care needs of paediatric palliative patients perceived by professionals: a grounded theory. Enferm Clin (Engl Ed).

[39] Page MJ, McKenzie JE, Bossuyt PM, Boutron I, Hoffmann TC, Mulrow CD (2021). The PRISMA 2020 statement: an updated guideline for reporting systematic reviews. BMJ.

[40] Siden H, Widger K. Research in children’s palliative care. In: Hain R, Goldman A, Rapoport A, Meiring M, Hain R, Goldman A, et al. editors. Oxford Textbook of Palliative Care for Children. Oxford University Press; 2021. p. 0.

[41] Ekberg S, Bowers A, Bradford N, Ekberg K, Rolfe M, Elvidge N (2022). Enhancing paediatric palliative care: a rapid review to inform continued development of care for children with life-limiting conditions. J Paediatr Child Health.

[42] Boyden JY, Feudtner C, Deatrick JA, Widger K, LaRagione G, Lord B (2021). Developing a family-reported measure of experiences with home-based pediatric palliative and hospice care: a multi-method, multi-stakeholder approach. BMC Palliat Care.

[43] Hökkä M, Martins Pereira S, Pölkki T, Kyngäs H, Hernández-Marrero P (2020). Nursing competencies across different levels of palliative care provision: a systematic integrative review with thematic synthesis. Palliat Med.

[44] Collier A, Hodgins M, Crawford G, Every A, Womsley K, Jeffs C (2019). What does it take to deliver brilliant home-based palliative care? Using positive organisational scholarship and video reflexive ethnography to explore the complexities of palliative care at home. Palliat Med.

[45] Beavis J, Davis L, McKenzie S (2021). Clinical Supervision for Support workers in Paediatric Palliative Care: A literature review. Clin Child Psychol Psychiatry.

[46] Greenfield K, Holley S, Schoth DE, Harrop E, Howard RF, Bayliss J (2020). A mixed-methods systematic review and meta-analysis of barriers and facilitators to paediatric symptom management at end of life. Palliat Med.

[47] Bradford N, Armfield NR, Young J, Smith AC (2013). The case for home based telehealth in pediatric palliative care: a systematic review. BMC Palliat Care.

[48] Holmen H, Riiser K, Winger A (2020). Home-based Pediatric Palliative Care and Electronic Health: systematic mixed methods review. J Med Internet Res.

[49] Miller KA, Baird J, Lira J, Herrera Eguizabal J, Fei S, Kysh L (2021). The Use of Telemedicine for Home-based Palliative Care for Children with Serious Illness: a scoping review. J Pain Symptom Manag.

