# Supplementary material for: The needs of healthcare personnel who provide home-based pediatric palliative care: a mixed method systematic review
Source: BMC Health Serv Res. 2024 Jan 9;24:45. doi: 10.1186/s12913-023-10495-7 (PMC10777650; doi:10.1186/s12913-023-10495-7)
Supplement: Supplementary file 1 — Supplementary Material 1 [file 12913_2023_10495_MOESM1_ESM.pdf]

Additional file 1. Search string

Database: Ovid MEDLINE(R) and Epub Ahead of Print, In-Process, In-Data-Review & Other Non-Indexed Citations and Daily 1946 to December 08, 2021
Dato: 09.12.2021
Treff: 1338

#	Searches	Results
1	Palliative Care/	58865
2	Advance Care Planning/	3601
3	Palliative Medicine/	449
4	"Hospice and Palliative Care Nursing"/	1564
5	Terminal Care/	30256
6	Terminally Ill/	6714
7	palliat*.tw,kw,kf.	85643
8	(advance* adj3 planning).tw,kw,kf.	5628
9	(advance* adj (disease* or illness* or stage*)).tw,kw,kf.	65176
10	(terminal adj (care or period*)).tw,kw,kf.	2621
11	((terminal* or incurabl* or irreversibl*) adj ill*).tw,kw,kf.	7931
12	((life limit* or life threatening) adj3 (disease* or condition* or illness*)).tw,kw,kf.	23380
13	(end of life or dying).tw,kw,kf.	59981
14	or/1-13	250949
15	Adolescent/	2143227
16	child/ or child, preschool/	2031635
17	infant/ or infant, newborn/ or infant, low birth weight/ or infant, small for gestational age/ or infant, very low birth weight/ or infant, extremely low birth weight/ or infant, postmature/ or infant, premature/ or infant, extremely premature/	1198441
18	Disabled Children/	6730
19	Pediatrics/	56806
20	puberty/	13837
21	Minors/	2706
22	(child* or Infan* or minors or pediatric* or paediatric* or newborn* or new born* or baby or babies or neonat* or kid or kids or toddler* or adoles* or preadoles* or teen* or boy* or girl* or underage* or under age* or juvenil* or youth* or puber* or schoolchild* or young people).tw,kw,kf.	2710858
23	or/15-22	4607601

24	Home Care Services/	35207
25	Home Nursing/	8638
26	Respite care/	1071
27	community health nursing/ or home health nursing/ or Community Mental Health Services/	38581
28	Hemodialysis, Home/	2049
29	home care services, hospital-based/ or home infusion therapy/ or parenteral nutrition, home/ or parenteral nutrition, home total/ or House Calls/	7861
30	home*.tw,kw,kf.	560132
31	Communit*.tw,kw,kf.	650029
32	(domiciliary or domestic* or dwelling* or residential).tw,kw,kf.	183318
33	or/24-32	1326570
34	exp Health Personnel/	566690
35	professional role/ or nurse's role/ or physician's role/	87493
36	((health or medical* or palliat*) adj2 (personnel or worker* or professional* or team*)).tw,kw,kf.	176367
37	(healthcare adj (personnel or worker* or professional* or team*)).tw,kw,kf.	49281
38	(nurse* or nursing).tw,kw,kf.	493774
39	(physical therapist* or physiotherapist*).tw,kw,kf.	16198
40	occupational therapist*.tw,kw,kf.	6289
41	(nutritionist* or dietician* or dietitian*).tw,kw,kf.	11503
42	(physician* or clinician*).tw,kw,kf.	665180
43	(pediatrician* or paediatrician*).tw,kw,kf.	24681
44	(psychologist* or psychiatrist*).tw,kw,kf.	41564
45	or/34-44	1678245
46	14 and 23 and 33 and 45	1338

Database: Embase 1974 to 2021 December 08 (Ovid)
Dato: 09.12.2021
Treff: 2383

#	Searches	Results
1	palliative therapy/	97535
2	palliative nursing/	1194
3	cancer palliative therapy/	25352
4	palliative chemotherapy/	722
5	advance care planning/	4220

6	terminal care/	38238
7	terminally ill patient/	8279
8	palliat*.tw,kw,kf.	134888
9	(advance* adj3 planning).tw,kw,kf.	8854
10	(advance* adj (disease* or illness* or stage*)).tw,kw,kf.	104721
11	(terminal adj (care or period*)).tw,kw,kf.	3544
12	((terminal* or incurabl* or irreversibl*) adj ill*).tw,kw,kf.	10248
13	((life limit* or life threatening) adj3 (disease* or condition* or illness*)).tw,kw,kf.	34098
14	(end of life or dying).tw,kw,kf.	81053
15	or/1-14	380537
16	adolescent/ or child/ or preschool child/ or infant/ or newborn/ or toddler/ or small for date infant/ or prematurity/ or pediatrics/ or school child/ or handicapped child/ or young adult/ or puberty/ or prepuberty/ or "minor (person)"/	3937480
17	(child* or Infan* or minors or pediatric* or paediatric* or newborn* or new born* or baby or babies or neonat* or kid or kids or toddler* or adoles* or preadoles* or teen* or boy* or girl* or underage* or under age* or juvenil* or youth* or puber* or schoolchild* or young people).tw,kw,kf.	3297034
18	or/16-17	4879824
19	home care/ or home dialysis/ or home health agency/ or home intravenous therapy/ or home mental health care/ or home monitoring/ or home oxygen therapy/ or home physiotherapy/ or home rehabilitation/ or home respiratory care/ or home visit/ or visiting nursing service/ or community health nursing/ or community psychiatric nursing/	98481
20	home*.tw,kw,kf.	742072
21	Communit*.tw,kw,kf.	793055
22	(domiciliary or domestic* or dwelling* or residential).tw,kw,kf.	213229
23	or/19-22	1652966
24	exp health care personnel/	1727682
25	exp medical personnel/	1055987
26	((health or medical* or palliat*) adj2 (personnel or worker* or professional* or team*)).tw,kw,kf.	226648
27	(healthcare adj (personnel or worker* or professional* or team*)).tw,kw,kf.	68986
28	(nurse* or nursing).tw,kw,kf.	561352
29	(physical therapist* or physiotherapist*).tw,kw,kf.	28360
30	occupational therapist*.tw,kw,kf.	9992
31	(nutritionist* or dietician* or dietitian*).tw,kw,kf.	20438

32	(physician* or clinician*).tw,kw,kf.	934972
33	(pediatrician* or paediatrician*).tw,kw,kf.	37045
34	(psychologist* or psychiatrist*).tw,kw,kf.	65190
35	or/24-34	2751360
36	15 and 18 and 23 and 35	2383

Database: APA PsycInfo 1806 to November Week 5 2021 (Ovid)
Dato: 09.12.2021
Treff: 1209

#	Searches	Results
1	palliative care/ or terminally ill patients/ or terminal cancer/ or "death and dying"/	47931
2	palliat*.tw.	13836
3	(advance* adj3 planning).tw.	1884
4	(advance* adj (disease* or illness* or stage*)).tw.	2690
5	(terminal adj (care or period*)).tw.	575
6	((terminal* or incurabl* or irreversibl*) adj ill*).tw.	4460
7	((life limit* or life threatening) adj3 (disease* or condition* or illness*)).tw.	3381
8	(end of life or dying).tw.	20347
9	or/1-8	63171
10	("100" or "120" or "140" or "160" or "180" or "200" or "320").ag.	1250853
11	child care/	8047
12	early adolescence/ or puberty/	5386
13	(child* or Infan* or minors or pediatric* or paediatric* or newborn* or new born* or baby or babies or neonat* or kid or kids or toddler* or adoles* or preadoles* or teen* or boy* or girl* or underage* or under age* or juvenil* or youth* or puber* or schoolchild* or young people).tw.	1089589
14	or/10-13	1669197
15	home care/ or home care personnel/ or homebound/ or outpatient treatment/ or home visiting programs/ or community mental health services/	24092
16	home*.tw.	174836
17	Communit*.tw.	323430
18	(domiciliary or domestic* or dwelling* or residential).tw.	73399
19	or/15-18	521105
20	exp Health Personnel/	174778

21	Clinicians/	11665
22	psychologists/	23367
23	psychiatrists/	12198
24	professional role/	623
25	((health or medical* or palliat*) adj2 (personnel or worker* or professional* or team*)).tw.	69527
26	(healthcare adj (personnel or worker* or professional* or team*)).tw.	10327
27	(nurse* or nursing).tw.	111037
28	(physical therapist* or physiotherapist*).tw.	2768
29	occupational therapist*.tw.	5643
30	(nutritionist* or dietician* or dietitian*).tw.	1520
31	(physician* or clinician*).tw.	161712
32	(pediatrician* or paediatrician*).tw.	5106
33	(psychologist* or psychiatrist*).tw.	126649
34	or/20-33	504814
35	9 and 14 and 19 and 34	1209

Database: Amed (Ovid)
Dato: 09.12.2021
Treff: 294

#	Searches	Results
1	palliative care/ or terminal care/ or terminal illness/	11670
2	palliat*.ti,ab.	9761
3	(advance* adj3 planning).ti,ab.	323
4	(advance* adj (disease* or illness* or stage*)).ti,ab.	505
5	(terminal adj (care or period*)).ti,ab.	289
6	((terminal* or incurabl* or irreversibl*) adj ill*).ti,ab.	1622
7	((life limit* or life threatening) adj3 (disease* or condition* or illness*)).ti,ab.	589
8	(end of life or dying).ti,ab.	5925
9	or/1-8	17401
10	adolescent/ or adolescence/ or child/ or child preschool/ or disabled children/ or infant/ or infant newborn/ or infant low birth weight/ or infant premature/ or Pediatrics/ or puberty/	24626

11	(child* or Infan* or minors or pediatric* or paediatric* or newborn* or new born* or baby or babies or neonat* or kid or kids or toddler* or adoles* or preadoles* or teen* or boy* or girl* or underage* or under age* or juvenil* or youth* or puber* or schoolchild* or young people).ti,ab.	59905
12	or/10-11	63826
13	community health services/ or community health nursing/ or home care services/ or Home nursing/ or community mental health services/	5499
14	home*.ti,ab.	14815
15	Communit*.ti,ab.	12784
16	(domiciliary or domestic* or dwelling* or residential).ti,ab.	3188
17	or/13-16	27548
18	exp health personnel/	6919
19	nurses role/ or physicians role/	967
20	((health or medical* or palliat*) adj2 (personnel or worker* or professional* or team*)).ti,ab.	4651
21	(healthcare adj (personnel or worker* or professional* or team*)).ti,ab.	774
22	(nurse* or nursing).ti,ab.	9667
23	(physical therapist* or physiotherapist*).ti,ab.	5509
24	occupational therapist*.ti,ab.	3968
25	(nutritionist* or dietician* or dietitian*).ti,ab.	156
26	(physician* or clinician*).ti,ab.	12127
27	(pediatrician* or paediatrician*).ti,ab.	143
28	(psychologist* or psychiatrist*).ti,ab.	758
29	or/18-28	36280
30	9 and 12 and 17 and 29	294

Database: Cinahl (Ebsco Host)
Dato: 10.12.2021
Treff: 891

#	Query	Results
S1	(MH "Palliative Care")	39,147
S2	(MH "Palliative Medicine")	50
S3	(MH "Hospice and Palliative Nursing")	5,393
S4	(MH "Terminal Care")	19,010
S5	(MH "Terminally Ill Patients")	11,901

S6	TI (palliat* OR (advance* N2 planning) OR (advance* N0 (disease* or illness* OR stage*)) OR (terminal N0 (care or period*)) OR ((terminal* OR incurabl* OR irreversibl*) N0 ill*) OR (("life limit*" or "life threatening") N2 (disease* or condition* or illness*)) OR "end of life" OR dying) OR AB (palliat* OR (advance* N2 planning) OR (advance* N0 (disease* or illness* OR stage*)) OR (terminal N0 (care or period*)) OR ((terminal* OR incurabl* OR irreversibl*) N0 ill*) OR (("life limit*" or "life threatening") N2 (disease* or condition* or illness*)) OR "end of life" OR dying)	90,204
S7	S1 OR S2 OR S3 OR S4 OR S5 OR S6	109,142
S8	(MH "Pediatrics")	20,902
S9	(MH "Child")	495,455
S10	(MH "Infant") OR (MH "Infant, Newborn") OR (MH "Infant, Large for Gestational Age") OR (MH "Infant, Low Birth Weight") OR (MH "Infant, Postmature") OR (MH "Infant, Premature")	271,784
S11	(MH "Child, Medically Fragile")	1,155
S12	(MH "Child, Disabled")	12,674
S13	(MH "Adolescence")	566,040
S14	(MH "Puberty") OR (MH "Minors (Legal)")	4,068
S15	TI ((child* or Infan* or Minors or pediatric* or paediatric* or newborn* or "new born*" or baby or babies or neonat* or kid or kids or toddler* or adoles* or preadoles* or teen* or boy* or girl* or underage* or "under age*" or juvenil* or youth* or puber* or schoolchild* or "young people")) OR AB ((child* or Infan* or minors or pediatric* or paediatric* or newborn* or "new born*" or baby or babies or neonat* or kid or kids or toddler* or adoles* or preadoles* or teen* or boy* or girl* or underage* or "under age*" or juvenil* or youth* or puber* or schoolchild* or "young people"))	944,553
S16	S8 OR S9 OR S10 OR S11 OR S12 OR S13 OR S14 OR S15	1,354,290
S17	(MH "Home Health Care")	24,468
S18	(MH "Home Apnea Monitoring")	85
S19	(MH "Home Dialysis") OR (MH "Home Intravenous Therapy") OR (MH "Home Nutritional Support") OR (MH "Home Respiratory Care") OR (MH "Home Rehabilitation") OR (MH "Home Occupational Therapy") OR (MH "Home Physical Therapy") OR (MH "Psychiatric Home Care") OR (MH "Home Oxygen Therapy")	7,200
S20	(MH "Home Nursing") OR (MH "Respite Care") OR (MH "Community Health Nursing") OR (MH "Home nursing, professional")	40,402
S21	(MH "Home Health Agencies")	5,230
S22	(MH "Home Environment")	11,606
S23	(MH "Home Visits")	6,534
S24	TI (Home* or Communit* or Domiciliary or Domestic* or dwelling* or residential) OR AB (Home* or Communit* or Domiciliary or Domestic* or dwelling* or residential)	478,741
S25	S17 OR S18 OR S19 OR S20 OR S21 OR S22 OR S23 OR S24	517,002
S26	(MH "Health Personnel+") OR (MH "Psychologists") OR (MH "Nursing Role") OR (MH "Physician's Role") OR (MH "Professional Role")	670,147
S27	TI (((health or medical* or palliat*) N1 (personnel or worker* or professional* or team*)) or (healthcare N0 (personnel or worker* or professional* or team*)) or nurse* or nursing or	928,112

	"physical therapist*" or physiotherapist* or "occupational therapist*" or nutritionist* or dietician* or dietitian* or physician* or clinician* or pediatrician* or paediatrician* or psychologist* or psychiatrist*) OR AB (((health or medical* or palliat*) N1 (personnel or worker* or professional* or team*)) or (healthcare N0 (personnel or worker* or professional* or team*)) or nurse* or nursing or "physical therapist*" or physiotherapist* or "occupational therapist*" or nutritionist* or dietician* or dietitian* or physician* or clinician* or pediatrician* or paediatrician*or psychologist* or psychiatrist*)	
S28	S26 OR S27	1,317,282
S29	S7 AND S16 AND S25 AND S28	891

Database: Cochrane Library
Dato: 10.12.2021
Treff: 81 (8 reviews, 73 trials)

ID	Search	Hits
#1	MeSH descriptor: [Palliative Care] this term only	1728
#2	MeSH descriptor: [Hospice and Palliative Care Nursing] this term only	34
#3	MeSH descriptor: [Advance Care Planning] this term only	199
#4	MeSH descriptor: [Palliative Medicine] this term only	2
#5	MeSH descriptor: [Terminal Care] this term only	374
#6	MeSH descriptor: [Terminally Ill] this term only	94
#7	(palliat* OR (advance* NEAR/3 planning) OR (advance* NEXT (disease* or illness* OR stage*)) OR (terminal NEXT (care or period*)) OR ((terminal* OR incurabl* OR irreversibl*) NEXT ill*) OR ((life NEXT limit* or "life threatening") NEAR/3 (disease* or condition* or illness*)) OR "end of life" OR dying):ti,ab,kw	17510
#8	{OR #1-#7}	17509
#9	MeSH descriptor: [Adolescent] this term only	108631
#10	MeSH descriptor: [Child] this term only	52041
#11	MeSH descriptor: [Child, Preschool] this term only	30881
#12	MeSH descriptor: [Infant] this term only	22963
#13	MeSH descriptor: [Infant, Newborn] this term only	16837
#14	MeSH descriptor: [Infant, Low Birth Weight] this term only	1039
#15	MeSH descriptor: [Infant, Small for Gestational Age] this term only	289
#16	MeSH descriptor: [Infant, Very Low Birth Weight] this term only	902
#17	MeSH descriptor: [Infant, Extremely Low Birth Weight] this term only	127
#18	MeSH descriptor: [Infant, Postmature] this term only	10
#19	MeSH descriptor: [Infant, Premature] this term only	3875
#20	MeSH descriptor: [Infant, Extremely Premature] this term only	225

#21	MeSH descriptor: [Disabled Children] this term only	122
#22	[mh ^pediatrics] OR [mh ^puberty] OR [mh ^Minors]	980
#23	(child* or Infan* or minors or pediatric* or paediatric* or newborn* or new NEXT born* or baby or babies or neonat* or kid or kids or toddler* or adoles* or preadoles* or teen* or boy* or girl* or underage* or under NEXT age* or juvenil* or youth* or puber* or schoolchild* or "young people"):ti,ab,kw	318507
#24	{OR #9-#23}	318509
#25	[mh ^"Home Care Services"] OR [mh ^"Home Nursing"] OR [mh ^"Respite care"] OR [mh ^"community health nursing"] OR [mh ^"Community Mental Health Services"] OR [mh ^"home health nursing"] OR [mh ^"Hemodialysis, Home"]	3210
#26	[mh ^"home care services, hospital-based"] OR [mh ^"home infusion therapy"] OR [mh ^"parenteral nutrition, home"] OR [mh ^"parenteral nutrition, home total"] OR [mh ^"House Calls"]	867
#27	(Home* or Communit* or Domiciliary or Domestic* or dwelling* or residential):ti,ab,kw	109689
#28	{OR #25-#27}	109714
#29	MeSH descriptor: [Health Personnel] explode all trees	9944
#30	[mh ^"professional role"] OR [mh ^"nurse's role"] OR [mh ^"physician's role"]	773
#31	(((health or medical* or palliat*) NEAR/2 (personnel or worker* or professional* or team*)) or (healthcare NEXT (personnel or worker* or professional* or team*)) or nurse* or nursing or physical NEXT therapist* or physiotherapist* or occupational NEXT therapist* or nutritionist* or dietician* or dietitian* or physician* or clinician* or pediatrician* or paediatrician* or psychologist* or psychiatrist*):ti,ab,kw	135376
#32	{OR #29-#31}	138078
#33	#8 AND #24 AND #28 AND #32	83

Database: Web of Science Core Collection (1987-present)
Dato: 10.12.2021
Treff: 1361

TS=(palliat* OR (advance* NEAR/2 planning) OR (advance* NEAR/0 (disease* or illness* OR stage*)) OR (terminal NEAR/0 (care or period*)) OR ((terminal* OR incurabl* OR irreversibl*) NEAR/0 (ill*)) OR (("life limit*" or "life threatening") NEAR/3 (disease* or condition* or illness*)) OR "end of life" OR dying) AND TS=(child* or Infan* or minors or pediatric* or paediatric* or newborn* or "new born*" or baby or babies or neonat* or kid or kids or toddler* or adoles* or preadoles* or teen* or boy* or girl* or underage* or "under age*" or juvenil* or youth* or puber* or schoolchild* or "young people") AND TS=(Home* or Communit* or Domiciliary or Domestic* or dwelling* or residential) AND TS=(((health or medical* or palliat*) NEAR/1 (personnel or worker* or professional* or team*)) or (healthcare NEAR/0 (personnel or worker* or professional* or team*)) or nurse* or nursing or "physical therapist*" or physiotherapist* or "occupational therapist*" or nutritionist* or dietician* or dietitian* or physician* or clinician* or pediatrician* or paediatrician* or psychologist* or psychiatrist*)

Updated search December 2023

Database: Ovid MEDLINE(R) and Epub Ahead of Print, In-Process, In-Data-Review & Other Non-Indexed Citations and Daily 1946 to December 01, 2023
Dato: 05.12.23
Limits: 2021 - Current
Treff: 314

#	Searches	Results
1	Palliative Care/	63858
2	Advance Care Planning/	4316
3	Palliative Medicine/	544
4	"Hospice and Palliative Care Nursing"/	2501
5	Terminal Care/	32085
6	Terminally Ill/	6823
7	palliat*.tw,kw,kf.	96983
8	(advance* adj3 planning).tw,kw,kf.	7053
9	(advance* adj (disease* or illness* or stage*)).tw,kw,kf.	74066
10	(terminal adj (care or period*)).tw,kw,kf.	3020
11	((terminal* or incurabl* or irreversibl*) adj ill*).tw,kw,kf.	8470
12	((life limit* or life threatening) adj3 (disease* or condition* or illness*)).tw,kw,kf.	28390
13	(end of life or dying).tw,kw,kf.	67748
14	or/1-13	281797
15	Adolescent/	2228548
16	child/ or child, preschool/	2175454
17	infant/ or infant, newborn/ or infant, low birth weight/ or infant, small for gestational age/ or infant, very low birth weight/ or infant, extremely low birth weight/ or infant, postmature/ or infant, premature/ or infant, extremely premature/	1261766
18	Disabled Children/	7041
19	Pediatrics/	57990
20	puberty/	14434
21	Minors/	2836
22	(child* or Infan* or minors or pediatric* or paediatric* or newborn* or new born* or baby or babies or neonat* or kid or kids or toddler* or adoles* or preadoles* or teen* or boy* or girl* or underage* or under age* or juvenil* or youth* or puber* or schoolchild* or young people).tw,kw,kf.	2997509
23	or/15-22	4930782
24	Home Care Services/	36568
25	Home Nursing/	8668
26	Respite care/	1094
27	community health nursing/ or home health nursing/ or Community Mental Health Services/	38796
28	Hemodialysis, Home/	2186
29	home care services, hospital-based/ or home infusion therapy/ or parenteral nutrition, home/ or parenteral nutrition, home total/ or House Calls/	8241
30	home*.tw,kw,kf.	647406
31	Communit*.tw,kw,kf.	771379
32	(domiciliary or domestic* or dwelling* or residential).tw,kw,kf.	213245
33	or/24-32	1547197
34	exp Health Personnel/	621988
35	professional role/ or nurse's role/ or physician's role/	89248
36	((health or medical* or palliat*) adj2 (personnel or worker* or professional* or team*)).tw,kw,kf.	206768

37	(healthcare adj (personnel or worker* or professional* or team*)).tw,kw,kf.	69076
38	(nurse* or nursing).tw,kw,kf.	542239
39	(physical therapist* or physiotherapist*).tw,kw,kf.	19218
40	occupational therapist*.tw,kw,kf.	7285
41	(nutritionist* or dietician* or dietitian*).tw,kw,kf.	13476
42	(physician* or clinician*).tw,kw,kf.	757361
43	(pediatrician* or paediatrician*).tw,kw,kf.	27259
44	(psychologist* or psychiatrist*).tw,kw,kf.	46035
45	or/34-44	1879097
46	14 and 23 and 33 and 45	1536
47	limit 46 to yr="2021 -Current"	314

Database: Embase 1974 to 2023 December 01
Dato: 05.12.23
Limits: 2021 - Current
Treff: 617

#	Searches	Results
1	palliative therapy/	114731
2	palliative nursing/	1717
3	cancer palliative therapy/	27715
4	palliative chemotherapy/	2327
5	advance care planning/	6638
6	terminal care/	42807
7	terminally ill patient/	8772
8	palliat*.tw,kw,kf.	155718
9	(advance* adj3 planning).tw,kw,kf.	11798
10	(advance* adj (disease* or illness* or stage*)).tw,kw,kf.	120090
11	(terminal adj (care or period*)).tw,kw,kf.	4015
12	((terminal* or incurabl* or irreversibl*) adj ill*).tw,kw,kf.	11114
13	((life limit* or life threatening) adj3 (disease* or condition* or illness*)).tw,kw,kf.	41469
14	(end of life or dying).tw,kw,kf.	93884
15	or/1-14	439073
16	adolescent/ or child/ or preschool child/ or infant/ or newborn/ or toddler/ or small for date infant/ or prematurity/ or pediatrics/ or school child/ or handicapped child/ or young adult/ or puberty/ or prepuberty/ or "minor (person)"/	4394194
17	(child* or Infan* or minors or pediatric* or paediatric* or newborn* or new born* or baby or babies or neonat* or kid or kids or toddler* or adoles* or preadoles* or teen* or boy* or girl* or underage* or under age* or juvenil* or youth* or puber* or schoolchild* or young people).tw,kw,kf.	3708934
18	or/16-17	5438322
19	home care/ or home dialysis/ or home health agency/ or home intravenous therapy/ or home mental health care/ or home monitoring/ or home oxygen therapy/ or home physiotherapy/ or home rehabilitation/ or home respiratory care/ or home visit/ or visiting nursing service/ or community health nursing/ or community psychiatric nursing/	108264
20	home*.tw,kw,kf.	864878
21	Communit*.tw,kw,kf.	939674
22	(domiciliary or domestic* or dwelling* or residential).tw,kw,kf.	247849
23	or/19-22	1936137
24	exp health care personnel/	2006728
25	exp medical personnel/	1234272
26	((health or medical* or palliat*) adj2 (personnel or worker* or professional* or team*)).tw,kw,kf.	267319

27	(healthcare adj (personnel or worker* or professional* or team*)).tw,kw,kf.	94969
28	(nurse* or nursing).tw,kw,kf.	624797
29	(physical therapist* or physiotherapist*).tw,kw,kf.	33417
30	occupational therapist*.tw,kw,kf.	11528
31	(nutritionist* or dietician* or dietitian*).tw,kw,kf.	24647
32	(physician* or clinician*).tw,kw,kf.	1074331
33	(pediatrician* or paediatrician*).tw,kw,kf.	41711
34	(psychologist* or psychiatrist*).tw,kw,kf.	73617
35	or/24-34	3161254
36	15 and 18 and 23 and 35	2855
37	limit 36 to yr="2021 -Current"	617

Database: APA PsycInfo 1806 to November Week 3 2023
Dato: 05.12.23
Limits: 2021 - Current
Treff: 166

#	Searches	Results
1	palliative care/ or terminally ill patients/ or terminal cancer/ or "death and dying"/	53994
2	palliat*.tw.	15851
3	(advance* adj3 planning).tw.	2322
4	(advance* adj (disease* or illness* or stage*)).tw.	2971
5	(terminal adj (care or period*)).tw.	664
6	((terminal* or incurabl* or irreversibl*) adj ill*).tw.	4672
7	((life limit* or life threatening) adj3 (disease* or condition* or illness*)).tw.	3751
8	(end of life or dying).tw.	22471
9	or/1-8	70573
10	("100" or "120" or "140" or "160" or "180" or "200" or "320").ag.	1371187
11	child care/	8773
12	early adolescence/ or puberty/	6332
13	(child* or Infan* or minors or pediatric* or paediatric* or newborn* or new born* or baby or babies or neonat* or kid or kids or toddler* or adoles* or preadoles* or teen* or boy* or girl* or underage* or under age* or juvenil* or youth* or puber* or schoolchild* or young people).tw.	1173479
14	or/10-13	1818752
15	home care/ or home care personnel/ or homebound/ or outpatient treatment/ or home visiting programs/ or community mental health services/	26646
16	home*.tw.	192327
17	Communit*.tw.	360585
18	(domiciliary or domestic* or dwelling* or residential).tw.	80765
19	or/15-18	577445
20	exp Health Personnel/	195937
21	Clinicians/	13786
22	psychologists/	24682
23	psychiatrists/	12965
24	professional role/	899
25	((health or medical* or palliat*) adj2 (personnel or worker* or professional* or team*)).tw.	77806
26	(healthcare adj (personnel or worker* or professional* or team*)).tw.	14027
27	(nurse* or nursing).tw.	122190
28	(physical therapist* or physiotherapist*).tw.	3179
29	occupational therapist*.tw.	6167
30	(nutritionist* or dietician* or dietitian*).tw.	1715

31	(physician* or clinician*).tw.	176517
32	(pediatrician* or paediatrician*).tw.	5433
33	(psychologist* or psychiatrist*).tw.	133182
34	or/20-33	552137
35	9 and 14 and 19 and 34	1358
36	limit 35 to yr="2021 -Current"	166

Database: AMED (Allied and Complementary Medicine) 1985 to October 2023
Dato: 05.12.23
Limits: 2021 - Current
Treff: 12

#	Searches	Results
1	palliative care/ or terminal care/ or terminal illness/	11930
2	palliat*.ti,ab.	9982
3	(advance* adj3 planning).ti,ab.	345
4	(advance* adj (disease* or illness* or stage*)).ti,ab.	530
5	(terminal adj (care or period*)).ti,ab.	290
6	((terminal* or incurabl* or irreversibl*) adj ill*).ti,ab.	1641
7	((life limit* or life threatening) adj3 (disease* or condition* or illness*)).ti,ab.	614
8	(end of life or dying).ti,ab.	6091
9	or/1-8	17762
10	adolescent/ or adolescence/ or child/ or child preschool/ or disabled children/ or infant/ or infant newborn/ or infant low birth weight/ or infant premature/ or Pediatrics/ or puberty/	25854
11	(child* or Infan* or minors or pediatric* or paediatric* or newborn* or new born* or baby or babies or neonat* or kid or kids or toddler* or adoles* or preadoles* or teen* or boy* or girl* or underage* or under age* or juvenil* or youth* or puber* or schoolchild* or young people).ti,ab.	64602
12	or/10-11	68576
13	community health services/ or community health nursing/ or home care services/ or Home nursing/ or community mental health services/	5679
14	home*.ti,ab.	15624
15	Communit*.ti,ab.	13825
16	(domiciliary or domestic* or dwelling* or residential).ti,ab.	3464
17	or/13-16	29321
18	exp health personnel/	7319
19	nurses role/ or physicians role/	977
20	((health or medical* or palliat*) adj2 (personnel or worker* or professional* or team*)).ti,ab.	4934
21	(healthcare adj (personnel or worker* or professional* or team*)).ti,ab.	888
22	(nurse* or nursing).ti,ab.	10060
23	(physical therapist* or physiotherapist*).ti,ab.	5932
24	occupational therapist*.ti,ab.	4266
25	(nutritionist* or dietician* or dietitian*).ti,ab.	169
26	(physician* or clinician*).ti,ab.	13004
27	(pediatrician* or paediatrician*).ti,ab.	147
28	(psychologist* or psychiatrist*).ti,ab.	804
29	or/18-28	38462
30	9 and 12 and 17 and 29	306
31	limit 30 to yr="2021 -Current"	12

Database: Cinahl EBSCOhost
Dato: 05.12.23
Limit: 2021 - Current
Treff: 195

#	Query	Results
S1	(MH "Palliative Care")	42,538
S2	(MH "Palliative Medicine")	138
S3	(MH "Hospice Nursing") OR (MH "Palliative Care Nursing")	5,663
S4	(MH "Terminal Care")	20,095
S5	(MH "Terminally Ill Patients")	12,340
S6	TI (palliat* OR (advance* N2 planning) OR (advance* N0 (disease* or illness* OR stage*)) OR (terminal N0 (care or period*)) OR ((terminal* OR incurabl* OR irreversibl*) N0 ill*) OR (("life limit*" or "life threatening") N2 (disease* or condition* or illness*)) OR "end of life" OR dying) OR AB (palliat* OR (advance* N2 planning) OR (advance* N0 (disease* or illness* OR stage*)) OR (terminal N0 (care or period*)) OR ((terminal* OR incurabl* OR irreversibl*) N0 ill*) OR (("life limit*" or "life threatening") N2 (disease* or condition* or illness*)) OR "end of life" OR dying)	100,870
S7	S1 OR S2 OR S3 OR S4 OR S5 OR S6	120,033
S8	(MH "Pediatrics")	21,210
S9	(MH "Child")	526,973
S10	(MH "Infant") OR (MH "Infant, Newborn") OR (MH "Infant, Large for Gestational Age") OR (MH "Infant, Low Birth Weight") OR (MH "Infant, Postmature") OR (MH "Infant, Premature")	285,635
S11	(MH "Child, Medically Fragile")	1,254
S12	(MH "Children with Disabilities")	13,192
S13	(MH "Adolescence")	606,464
S14	(MH "Puberty") OR (MH "Minors (Legal)")	4,361
S15	TI ((child* or Infan* or Minors or pediatric* or paediatric* or newborn* or "new born*" or baby or babies or neonat* or kid or kids or toddler* or adoles* or preadoles* or teen* or boy* or girl* or underage* or "under age*" or juvenil* or youth* or puber* or schoolchild* or "young people")) OR AB ((child* or Infan* or minors or pediatric* or paediatric* or newborn* or "new born*" or baby or babies or neonat* or kid or kids or toddler* or adoles* or preadoles* or teen* or boy* or girl* or underage* or "under age*" or juvenil* or youth* or puber* or schoolchild* or "young people"))	1,055,109
S16	S8 OR S9 OR S10 OR S11 OR S12 OR S13 OR S14 OR S15	1,477,948
S17	(MH "Home Health Care")	25,886
S18	(MH "Home Apnea Monitoring")	85
S19	(MH "Home Dialysis") OR (MH "Home Intravenous Therapy") OR (MH "Home Nutritional Support") OR (MH "Home Respiratory Care") OR (MH "Home Rehabilitation") OR (MH "Home Occupational Therapy") OR (MH "Home Physical Therapy") OR (MH "Psychiatric Home Care") OR (MH "Home Oxygen Therapy")	7,589
S20	(MH "Home Nursing") OR (MH "Respite Care") OR (MH "Community Health Nursing") OR (MH "Home Health Nursing")	38,739
S21	(MH "Home Health Agencies")	5,239
S22	(MH "Home Environment")	12,659
S23	(MH "Home Visits")	6,681
S24	TI (Home* or Communit* or Domiciliary or Domestic* or dwelling* or residential) OR AB (Home* or Communit* or Domiciliary or Domestic* or dwelling* or residential)	534,627

S25	S17 OR S18 OR S19 OR S20 OR S21 OR S22 OR S23 OR S24	571,408
S26	(MH "Health Personnel+") OR (MH "Psychologists") OR (MH "Nursing Role") OR (MH "Physician's Role") OR (MH "Professional Role")	700,057
S27	TI (((health or medical* or palliat*) N1 (personnel or worker* or professional* or team*)) or (healthcare N0 (personnel or worker* or professional* or team*)) or nurse* or nursing or "physical therapist*" or physiotherapist* or "occupational therapist*" or nutritionist* or dietician* or dietitian* or physician* or clinician* or pediatrician* or paediatrician* or psychologist* or psychiatrist*) OR AB (((health or medical* or palliat*) N1 (personnel or worker* or professional* or team*)) or (healthcare N0 (personnel or worker* or professional* or team*)) or nurse* or nursing or "physical therapist*" or physiotherapist* or "occupational therapist*" or nutritionist* or dietician* or dietitian* or physician* or clinician* or pediatrician* or paediatrician*or psychologist* or psychiatrist*)	997,231
S28	S26 OR S27	1,389,539
S29	S7 AND S16 AND S25 AND S28	1,010
S30	S7 AND S16 AND S25 AND S28<br><br>Limiters - Publication Date: 20210101-20231231	195

Kommentar: Cinahl has deleted some of the subject headings since the last search and replaced them with new ones. This applies to:
Line 3: (MH "Hospice Nursing") OR (MH "Palliative Care Nursing") replace (MH "Hospice and Palliative Nursing")
Line 12: (MH "Children with Disabilities") replace (MH "Child, Disabled")
Line 20: (MH "Home Health Nursing") replace (MH "Home nursing, professional")

Database: Web of Science
Dato: 05.12.2023
Limits: 2021 - Current
Treff: 405

TS=(palliat* OR (advance* NEAR/2 planning) OR (advance* NEAR/0 (disease* or illness* OR stage*)) OR (terminal NEAR/0 (care or period*)) OR ((terminal* OR incurabl* OR irreversibl*) NEAR/0 (ill*)) OR (("life limit*" or "life threatening") NEAR/3 (disease* or condition* or illness*)) OR "end of life" OR dying) AND TS=(child* or Infan* or minors or pediatric* or paediatric* or newborn* or "new born*" or baby or babies or neonat* or kid or kids or toddler* or adoles* or preadoles* or teen* or boy* or girl* or underage* or "under age*" or juvenil* or youth* or puber* or schoolchild* or "young people") AND TS=(Home* or Communit* or Domiciliary or Domestic* or dwelling* or residential) AND TS=(((health or medical* or palliat*) NEAR/1 (personnel or worker* or professional* or team*)) or (healthcare NEAR/0 (personnel or worker* or professional* or team*)) or nurse* or nursing or "physical therapist*" or physiotherapist* or "occupational therapist*" or nutritionist* or dietician* or dietitian* or physician* or clinician* or pediatrician* or paediatrician* or psychologist* or psychiatrist*)

Database: Cochrane
Dato: 05.12.2023
Limits: 2021-2023
Treff: 15+2

ID	Search	Hits
#1	MeSH descriptor: [Palliative Care] this term only	2282
#2	MeSH descriptor: [Hospice and Palliative Care Nursing] this term only	78
#3	MeSH descriptor: [Advance Care Planning] this term only	329
#4	MeSH descriptor: [Palliative Medicine] this term only	4
#5	MeSH descriptor: [Terminal Care] this term only	510
#6	MeSH descriptor: [Terminally Ill] this term only	111
#7	(palliat* OR (advance* NEAR/3 planning) OR (advance* NEXT (disease* or illness* OR stage*)) OR (terminal NEXT (care or period*)) OR ((terminal* OR incurabl* OR irreversibl*) NEXT ill*) OR ((life NEXT limit* or "life threatening") NEAR/3 (disease* or condition* or illness*)) OR "end of life" OR dying):ti,ab,kw	20142
#8	{OR #1-#7}	20142
#9	MeSH descriptor: [Adolescent] this term only	126194
#10	MeSH descriptor: [Child] this term only	70232
#11	MeSH descriptor: [Child, Preschool] this term only	35374
#12	MeSH descriptor: [Infant] this term only	28999
#13	MeSH descriptor: [Infant, Newborn] this term only	20638
#14	MeSH descriptor: [Infant, Low Birth Weight] this term only	1173
#15	MeSH descriptor: [Infant, Small for Gestational Age] this term only	361
#16	MeSH descriptor: [Infant, Very Low Birth Weight] this term only	1069
#17	MeSH descriptor: [Infant, Extremely Low Birth Weight] this term only	152
#18	MeSH descriptor: [Infant, Postmature] this term only	10
#19	MeSH descriptor: [Infant, Premature] this term only	4685
#20	MeSH descriptor: [Infant, Extremely Premature] this term only	336
#21	MeSH descriptor: [Disabled Children] this term only	153
#22	[mh ^pediatrics] OR [mh ^puberty] OR [mh ^Minors]	1481
#23	(child* or Infan* or minors or pediatric* or paediatric* or newborn* or new NEXT born* or baby or babies or neonat* or kid or kids or toddler* or adoles* or preadoles* or teen* or boy* or girl* or underage* or under NEXT age* or juvenil* or youth* or puber* or schoolchild* or "young people"):ti,ab,kw	362448
#24	{OR #9-#23}	362448
#25	[mh ^"Home Care Services"] OR [mh ^"Home Nursing"] OR [mh ^"Respite care"] OR [mh ^"community health nursing"] OR [mh ^"Community Mental Health Services"] OR [mh ^"home health nursing"] OR [mh ^"Hemodialysis, Home"]	3606
#26	[mh ^"home care services, hospital-based"] OR [mh ^"home infusion therapy"] OR [mh ^"parenteral nutrition, home"] OR [mh ^"parenteral nutrition, home total"] OR [mh ^"House Calls"]	1014
#27	(Home* or Communit* or Domiciliary or Domestic* or dwelling* or residential):ti,ab,kw	130060
#28	{OR #25-#27}	130088
#29	MeSH descriptor: [Health Personnel] explode all trees	13585
#30	[mh ^"professional role"] OR [mh ^"nurse's role"] OR [mh ^"physician's role"]	1101
#31	(((health or medical* or palliat*) NEAR/2 (personnel or worker* or professional* or team*)) or (healthcare NEXT (personnel or worker* or professional* or team*)) or nurse* or nursing or	162567

	physical NEXT therapist* or physiotherapist* or occupational NEXT therapist* or nutritionist* or dietician* or dietitian* or physician* or clinician* or pediatrician* or paediatrician* or psychologist* or psychiatrist*):ti,ab,kw	
#32	{OR #29-#31}	166449
#33	#8 AND #24 AND #28 AND #32 with Publication Year from 2021 to 2023, in Trials	15
	Cochrane reviews 2021-2023	2
