# Supplementary material for: The needs of healthcare personnel who provide home-based pediatric palliative care: a mixed method systematic review
Source: BMC Health Serv Res. 2024 Jan 9;24:45. doi: 10.1186/s12913-023-10495-7 (PMC10777650; doi:10.1186/s12913-023-10495-7)
Supplement: Supplementary file 2 — Supplementary Material 2 [file 12913_2023_10495_MOESM2_ESM.pdf]

**Additional file 2.** Characteristics of the included studies (n = 21)

| [Study] author(s) and year | Country | Sample | Phenomenon of interest | Design | Evaluation | Results |
|---|---|---|---|---|---|---|
| **[19] Bertrand, A., Veyet, V., Goy, F., Cervos, M., & Schell, M. (2021)** | France | Home nurses (n = 25) | To describe how home nurses experienced providing palliative pediatric care in collaboration with a Home Care Unit (HCU) and analyze practices in a home-based palliative setting. | Retrospective, observational, single-center study | The self-administered questionnaires consisted of 6 parts: nurse and patients' demographic data, felt before home care implementation, during home care, and after patient's death and were analyzed with descriptive statistic. | Some home nurses had never provided pediatric palliative care. Many of the home nurses acknowledged having misgivings accepting these duties. The factors that facilitated providing this care were the availability of the HCU doctor and nurses, the proactiveness of the HCU team, and house calls. In many cases, the involvement of the home nurses exceeded the strictly professional setting. Half of the home nurses were amenable to undertaking another pediatric palliative care engagement, although half of the home nurses deemed the remuneration to be somewhat lacking. |
| **[20] Bradford, N. K., Young, J., Armfield, N. R., Herbert, A., & Smith, A. C. (2014)** | Australia | Medical (n = 4)<br>Nursing (n = 5)<br>Allied health (n = 1) | To investigate the barriers, enablers, and perceived usefulness for an established home telehealth program in pediatric palliative care from the perspective of clinicians. | Qualitative interview study | Iterative analysis was used to thematically analyze data and identify themes and core concepts from interviews. | Four themes are reported: managing relationships; expectations of clinicians; co-ordination, and the telehealth compromise. Core concepts that emerged from the data were the perceived ability to control clinical encounters in a virtual environment and the need to trust technology. These concepts help explain the telehealth compromise and low utilization of the home telehealth program. |

| [Study] author(s) and year | Country | Sample | Phenomenon of interest | Design | Evaluation | Results |
|---|---|---|---|---|---|---|
| **[21] Brenner, M., Connolly, M., Cawley, D., Howlin, F., Berry, J., & Quinn, C. (2016)** | Ireland | Parents (n = 7) Multi-disciplinary stakeholders including specialist nursing services, medicine, and allied healthcare professionals (n = 18) In-patient hospice staff (n = 13) Hospice at home team (n = 8) | To understand the experiences of families receiving a nurse led pilot hospice at home program and the experiences of healthcare professionals delivering and engaging with the program. | Qualitative interview study | A qualitative descriptive approach was used to analyze the data. | The findings from healthcare professionals centered on communication within and across services, education and training and lone working. |
| **[22] Campbell, L. M., & Amin, N. (2013)** | South Africa | Nurses (n = 5) Home-based care workers (n = 8) | To examine experiences of providers of palliative care to children when they attempted to fulfil one of their roles as palliative caregivers. | Discussions with participants, using photographs taken by the caregivers to stimulate and contextualize the discussions. | The analysis involved looking at themes that emerged from the data rather than matching data to predetermined categories. Analysis involved five steps: familiarization and immersion in the text; generating themes; coding; elaboration; and interpretation and checking. | Participants experienced four dilemmas regarding telling bad news: when families did not want to be told any bad news; when participants felt uncomfortable about telling bad news; when participants and patients shared dissimilar values about telling bad news; and when participants were unsure about when to tell bad news. |

| [Study] author(s) and year | Country | Sample | Phenomenon of interest | Design | Evaluation | Results |
|---|---|---|---|---|---|---|
| **[23] Castor, C., Hallström, I., Hansson, H., & Landgren, K. (2017)** | Sweden | Nurses (n=24), Counselors (n=4) Physicians (n=3) Assistant nurses (n=2) Occupational therapist (n=1) Physiotherapist (n=1) Dietitian (n=1) | To explore healthcare professionals' conceptions of caring for sick children in home care services. | Qualitative interview study | Data were analyzed stepwise using a phenomenographic analysis. | Three description categories emerged: "A challenging opportunity", "A child perspective", and "Reorganize in accordance with new prerequisites." Providing home care services for children was conceived to evoke both professional and personal challenges such as feelings of inadequacy and fear and professional growth such as increased competence and satisfaction. Conceptions of whether the home or the hospital was the best place for care differed. Adapting to the child's care was conceived as important. Cooperation with pediatric departments and a well-functioning teamwork were important organizational aspects. |
| **[24] Chong, L., & Abdullah, A. (2017)** | Malaysia | Nurses (n = 16) | To explore the experience of community palliative care nurses providing home care to children. | Qualitative interview study | Data were analyzed with thematic analysis. | Two categories were identified: challenges nurses faced and coping strategies. The themes identified from the categories are communication challenges, inadequate training and knowledge, personal suffering, challenges of the system, intrapersonal coping skills, interpersonal coping strategies, and systemic supports. |

| [Study] author(s) and year | Country | Sample | Phenomenon of interest | Design | Evaluation | Results |
|---|---|---|---|---|---|---|
| **[25] Kremeike K., Eulitz N., Junger S., Sander A., Geraedts M., & Reinhardt D. (2012)** | Germany | General pediatricians (n = 141) | To evaluate involvement in and contribution of general pediatricians in pediatric palliative care and their cooperation with other pediatric palliative care providers. | Survey research | A standardized questionnaire was designed based on an established instrument used in North-Rhine Westphalia. | The most general pediatricians stated that they had professional experience with pediatric palliative care. Collaboration of general pediatricians and other palliative care providers was stated as not well developed. The support by a specialized team including 24-hour on-call duty and the intensification of educational programs were emphasized. |
| **[26] Neilson S., Gibson F., Jeffares S., & Greenfield SM. (2020)** | UK | General practitioners (n = 32) | To examine general practitioners' perception of their role in children's oncology palliative care. | Mixed-methods study | Q methodology - a research method that clusters participants' experiences according to similar viewpoints. | Shared viewpoints were identified denoting different roles: the compassionate practitioner, the team player practitioner, and the pragmatic practitioner. In addition consensus (time pressures, knowledge deficits, emotional toll) and disagreement (psychological support, role, experiential learning, prior relationships) between the viewpoints were identified and examined. |
| **[27] Neilson, S. J., Kai, J., McArthur, C., & Greenfield, S. (2013)** | UK | Pediatric nurses (n = unknown) | To explore influences on the experiences of pediatric nurses providing out of hours palliative care within the family home to children with cancer. | Qualitative interview study | Data were analyzed following a grounded theory Chronological comparative data analysis identifying generated themes. Social worlds theory was used as a framework to examine the data. | Nurses' experiences are shaped by their social world and those of the nursing team, child and family and the inter-professional team providing the care. The lack of a formalized service, sub-optimal inter-professional working and impact of social worlds influence the experience of the nurse. |

| [Study] author(s) and year | Country | Sample | Phenomenon of interest | Design | Evaluation | Results |
|---|---|---|---|---|---|---|
| **[28] Porter, A. S., Zalud, K., Applegarth, J., Woods, C., Gattas, M., Rutt, E., Williams, K., Baker, J. N., & Kaye, E. C. (2021)** | United States | Hospice nurses (n = 41) | To assess the pediatric-specific training and support needs of hospice nurses caring for children in the community. | Qualitative interview study | Data were analyzed with content analysis. | Hospice nurses reported feeling uncomfortable caring for children with serious illness, and all nurses used language to express the immediacy behind the need for pediatric specific training and support. Nurses explained why further training and support are needed and clear preferences for who should provide the education, educational modalities, and specific training topics. Nurses also articulated barriers to training and support opportunities and proposed innovative suggestions for overcoming these challenges. Nurses emphasized the need for connection with experts, a sense of community, and solidarity to support frontline clinicians providing end-of-life care to children in the community. |
| **[29] Reid, F. C. (2013)** | Scotland | Nurses (n = 7) | To explore nurses lived experiences regarding the personal, contextual and interprofessional challenges faced when delivering palliative and end of life care to children and young people in the community. | Qualitative interview study | Data were analyzed thematically, using a coding, weighting, and distillation approach. | Four themes emerged: service delivery, nurse-family relationships, nurses' grief, funeral rites, and bereavement support. Nurses experienced considerable internal and external pressures. Some are inevitable but others, such as organization of care provision to families and nurses' personal coping, could be improved by adequately resourced workforces, integrated service structures and guidance on reflective practice. |

| [Study] author(s) and year | Country | Sample | Phenomenon of interest | Design | Evaluation | Results |
|---|---|---|---|---|---|---|
| **[30] Reid, F. C. (2013)** | Scotland | Adult community nurses (n = 10) | To explore challenges perceived by rural adult community nurses when delivering palliative care to children and young person and their families in the home. | Qualitative interview study | Data were analyzed using a qualitative phenomenological thematic approach. | Four key themes emerged: emotional preparedness, navigating the professional 'road', becoming part of the family, and it's everybody's business. |
| **[31] van der Geest, I. M. M., Bindels, P. J. E., Pluijm, S. M. F., Michiels, E. M. C., van der Heide, A., Pieters, R., Darlington, A. S. E., & van den Heuvel-Eibrink, M. M. (2017)** | The Nether-lands | General practitioners (GPs) (n = 91) | To explore the perspectives of general practitioners who care for children with advanced-stage cancer in a home-based setting | Cross-sectional study | Questionnaire addressing perspectives regarding: 1) symptom management, 2) collaboration with other health care professionals, 3) the child's death and care after death, and 4) impact of having provided palliative care, scored on distress thermometer (range 0-10). | The most prevalent symptoms reported in the patients were fatigue and pain. Difficulties with communicating, with coordinating, with collaborating and with contacting fellow members of the multidisciplinary treatment team were rare. Hectic and shocking situations and panic around the child's death were rare. GPs reported feelings of sadness and/or powerlessness around the time of the patient's death, and they rated their own distress level as relatively high during the terminal phase. Most GPs reported that they ultimately came to terms with the child's death. |

| [Study] author(s) and year | Country | Sample | Phenomenon of interest | Design | Evaluation | Results |
|---|---|---|---|---|---|---|
| **[32] Verberne L. M., Kars M. C., Schepers S. A., Schouten-Van Meeteren A. Y. N., Grootenhuis M. A., & Van Delden J. JM.** | The Nether-lands | Doctors (n = 20)<br>Nurses (n = 33)<br>Paramedics (n = 7)<br>Psychosocial professionals (n = 9)<br>Others (n = 2) | To identify barriers and facilitators reported by healthcare professionals (HCP`s) in primary, secondary or tertiary care for implementing a newly initiated multidisciplinary pediatric palliative care team (PPCT). | Cross-sectional study | An adjusted version of the questionnaire Measurement Instrument for Determinants of Innovations with additional open-ended questions. | Reported barriers to implementing a PPCT were related to the HCP`s own organization.<br>Reported facilitators were mainly related to the intervention and the user scale and only once to the organization scale. Additionally, HCPs expressed the need for clarity about tasks of the PPCT and reported having made a transition from feeling threatened by the PPCT to satisfaction about the PPCT. |
| **[33] Vollenbroich, R., Duroux, A., Grasser, M., Brandstätter, M., Borasio, G. D., & Führer, M. (2012)** | Germany | Parent dyads (n = 43)<br>Physicians (n = 54)<br>Nurses (n = 28)<br>Social workers (n = 8)<br>Psychologists (n = 5)<br>Physiotherapists (n = 5)<br>Chaplains (n = 3)<br>Case managers (n = 2) | To assess the effectiveness of a specialized pediatric palliative home care team (PPHCT) as experienced by parents and health care professionals. | Survey research | Questionnaire for health care professionals consisting of 22 items numerical rating scale (1-10) questions on quality of care and communication before and after PPHCT involvement. In addition, space for open comments was provided. | Healthcare professionals evaluated all investigated care domains (particularly cooperation/ communication/family support) as being significantly improved.<br>Thirty-five percent of Health care professionals felt uncertain concerning pediatric palliative care; and would welcome specific training opportunities. |

| [Study] author(s) and year | Country | Sample | Phenomenon of interest | Design | Evaluation | Results |
|---|---|---|---|---|---|---|
| **[34] Wallace, E., Twomey, M., & O'Reilly, M. (2012)** | Ireland | General practitioners (GPs) (n = 20) | To explore the experiences of general practitioners caring for children with central venous access devices in the community to establish barriers to their use and to assess methods of addressing these challenges. | Survey research | A standardized questionnaire containing 14 questions, with an opportunity for an additional free text response, was designed. | Thirteen GPs had no education in central venous access devices management and 14 were unaware of existing guidelines. The main challenges identified by GPs were lack of education, line management difficulties, infection risk, infrequent exposure to central venous access devices, and poor communication. GPs felt that these challenges could be addressed through education, increased manpower, community support, and improved communication. |
| **[35] Weaver, M. S., Neumann, M. L., Navaneethan, H., Robinson, J. E., & Hinds, P. S. (2020)** | United States | Hospice nurses (n = 15) | To explore the experiences of rural hospice nurses caring forchildren at the end of life using telehealth modalities to inform palliative communication. | Qualitative interview study | Data were analyzed with semantic content analysis. | Five themes about telehealth emerged: accessible support, participant inclusion, timely communication, informed and trusted planning, and familiarity fostered. Each theme had both benefits and cautions associated as well as telehealth suggestions. Nurses recommended individualizing communication, pacing content, fostering human connection, and developing relationships even with technology use. |
| **[36] Rico-Mena, P., Güeita-Rodríguez, J., Martino-Alba, R., Castel-Sánchez, M., Palacios-Ceña, D. (2023)** | Spain | Specialist Palliative Care Doctor (n = 7), Specialist Palliative Care Nurse (n = 5), Specialist Palliative Care Psychologist (n = 2), Specialist Palliative Care Social | To describe the feelings and emotions of professionals working in an interdisciplinary pediatric palliative home care team. | Qualitative interview study | Data were analyzed with thematic analysis. | Two themes emerged: (a) changing life for the better, which described how professionals value life more and helping children and families provides compassion satisfaction, which is comforting and explains their dedication to care; (b) adverse effects of work highlighted |

| [Study] author(s) and year | Country | Sample | Phenomenon of interest | Design | Evaluation | Results |
|---|---|---|---|---|---|---|
| | | Worker (n = 2), Specialist Palliative Care Physiotherapist (n = 1), Specialist Palliative Care Administrative Staff (n = 1) | | | | the emotional burden of caring for children with life-limiting or life-threatening illnesses, which can affect their job satisfaction and may lead to burnout, showing how experiencing in-hospital child deaths with suffering leads professionals to develop an interest in specializing in pediatric palliative care. |
| **[37] Larsen, S., Bording, I., Bjergegaard, M., Buchreitz, J., Mouritzen, B., Brix, L., Jespersen, B., Neergaard, M. (2023)** | Denmark | Primary care nurses (n = 20) | investigate how primary care nurses evaluated a shared care model between a specialized pediatrics palliative care team and primary care nurses in pediatric end-of-life care. | Survey research | 23-item questionnaire including: demographic questions, questions to evaluate the shared care model and, general job-satisfaction | The primary care nurses felt better prepared for pediatric end-of-life care, felt better at dealing with their own emotions and at cooperating in the local team. The majority felt the model helped coping with parents' pressure according to expectations and demands. The possibility of telephone contact and meetings with a specialized pediatric palliative care team in the patients' homes made them feel supported during the trajectory. The great majority of the primary care nurses stated that the trajectory changed how they saw themselves being involved in future pediatric palliative care. |
| **[38] Rico-Mena, P., Güeita-Rodríguez, J., Martino-Alba, R., Chocarro-Gonzalez, L., Sanz-Esteban, I.; Palacios-Ceña, D. (2023)** | Spain | Pediatrician (n = 7)<br>Nurse (n = 5)<br>Psychologist (n = 2)<br>Social worker (n = 2)<br>Physical therapist (n = 1)<br>Administrative Staff (n = 1) | Describe the process of delivery of pediatric palliative care from the perspective of a pediatric interdisciplinary | Qualitative descriptive case study<br><br>(semi-structured interviews, focus groups | Data were analyzed with inductive thematic analysis. | Two themes emerged: a) Pediatric palliative care is associated with life and represents provision of special care in highly complex children, in the context of the home, far from the hospital environment, and b) the team is key, the interdisciplinary nature of the team |

| [Study] author(s) and year | Country | Sample | Phenomenon of interest | Design | Evaluation | Results |
|---|---|---|---|---|---|---|
| | | Physiatrist (n = 3)<br>Neuro-pediatrician (n = 1)<br>Parents (n = 6) | team and the children's parents. | and field notes) | | provides a more comprehensive view of the child and family, fosters communication, and improves coordination. |
| **[39] Santana-Medina, J., Rodríguez-Suárez, C. A. (2023)** | Spain | Pediatric nurses (n = 8)<br>Pediatrician (n = 8)<br>Social worker (n = 2) | Explore the elements involved in the process of pediatric palliative home care in the Spanish context according to the opinion of professionals. | Qualitative in-depth interview study | Data were analyzed based on an inductive grounded theory approach | Data were analyzed and structured in four thematic groups (care, environment, patient and family, and professionals). The findings showed a holistic view emphasizing the need to organize and integrate the factors involved in the home-based approach to pediatric palliative home care. |
