# Supplementary material for: The needs of healthcare personnel who provide home-based pediatric palliative care: a mixed method systematic review
Source: BMC Health Serv Res. 2024 Jan 9;24:45. doi: 10.1186/s12913-023-10495-7 (PMC10777650; doi:10.1186/s12913-023-10495-7)
Supplement: Supplementary file 3 — Supplementary Material 3 [file 12913_2023_10495_MOESM3_ESM.pdf]

Additional file 3. Quality assessment

Category of study design	Qualitative studies													Quantitative descriptive studies							Mixed method study
	Bradford et al. (20)	Brenner et al. (21)	Campbell & Amin (22)	Castor et al. (23)	Chong & Abdullah (24)	Neilson et al. (27)	Porter et al. (28)	Reid (29)	Reid (30)	Weaver et al. (35)	Rico-Mena et al. (36)	Rico-Mena et al. (38)	Santana-Medina; Rodríguez-Suárez (39)	Bertrand et al. (19)	Kremeike et al. (25)	van der Geest et al. (31)	Verberne et al. (32)	Vollenbroich et al. (33)	Wallace et al. (34)	Larsen et al. (37)	Neilson et al. (26)
**Screening questions for all types**																					
Are there clear research questions?	Y	Y	Y	Y	Y	Y	Y	Y	Y	Y	Y	Y	Y	Y	Y	Y	Y	Y	Y	Y	Y
Do the collected data allow to adress the research question?	Y	Y	Y	Y	Y	Y	Y	Y	Y	Y	Y	Y	Y	Y	Y	Y	Y	Y	Y	Y	Y
**MMA1 Qualitative**																					
Is the qualitative approach appropriate to answer the research question?	Y	Y	Y	Y	Y	Y	Y	Y	Y	Y	Y	Y	Y								Y
Are the qualitative data collection methods adequate to address the research question?	Y	Y	?	Y	Y	Y	Y	Y	Y	Y	Y	Y	Y								Y
Are the findings adequately derived from the data?	Y	Y	Y	Y	Y	Y	Y	Y	Y	Y	Y	Y	Y								?
Is the interpretation of results sufficiently substantiated by data?	Y	?	Y	Y	Y	Y	Y	Y	Y	Y	Y	Y	Y								?
Is there coherence between qualitative data sources, collection, analysis and interpretation?	Y	?	?	Y	Y	Y	Y	Y	Y	Y	Y	Y	Y								?
**MMA4 Quantitative descriptive**																					
Is the sampling strategy relevant to address the research question?														Y	Y	Y	Y	Y	Y	Y	Y
Is the sample representative of the target population?														Y	Y	Y	?	Y	?	?	?
Are the measurements appropriate?														?	?	Y	Y	Y	?	Y	Y
Is the risk of nonresponse bias low?														N	?	Y	?	?	?	?	?
Is the statistical analysis appropriate to answer the research question?														Y	Y	Y	Y	Y	?	Y	Y
**MMA5 Mixed methods**																					
Is there an adequate rationale for using a mixed methods design to address the research question?																					Y
Are the different components of the study effectively integrated to answer the research question?																					Y
Are the outputs of the integration of qualitative and quantitative components adequately interpreted?																					?
Are divergences and inconsistencies between quantitative and qualitative results adequately addressed?																					?
Do the different components of the study adhere to the quality criteria of each tradition of the methods involved?																					?

Yes	Y
No	N
Can`t tell	?
