# Supplementary material for: The needs of healthcare personnel who provide home-based pediatric palliative care: a mixed method systematic review
Source: BMC Health Serv Res. 2024 Jan 9;24:45. doi: 10.1186/s12913-023-10495-7 (PMC10777650; doi:10.1186/s12913-023-10495-7)
Supplement: Supplementary file 4 — Supplementary Material 4 [file 12913_2023_10495_MOESM4_ESM.pdf]

Additional fil 4. Examples of transformed quantitative data into "qualitized" data and coded as needs

Quantitative data	Transformed "qualitized" data	Results coded as needs
The only facilitator in the organisation scale was 'information accessibility': 80% of the healthcare professionals reported that they easily received information about patients/parents from the paediatric palliative care team (31).	The primary facilitator at the organizational level was 'information accessibility,' with most healthcare professionals reporting that they consistently received information about patients/parents from the pediatric palliative care team (31).	Healthcare personnel's need for information
The general practitioners (GPs) reported that, among others, they received support for coming to terms with the patient's death from his/ her own family members (n = 61, 70%), fellow GPs (n = 40, 46%), and/or friends (n = 15, 17%) (30)	To process the patient's death, a little more than half of the GPs sought support from their families, some sought support from other GPs and a few GPs sought support from friends.	Healthcare personnel's need for support from others in coping with the patient's death
Education in basic palliative medicine was favoured by 119 (84.4%) and a sufficient information exchange with other care providers by 116 (82.3%) of the general paediatricians (24)	Most of the general pediatricians favored education in basic palliative medicine and sufficient information exchange with other care providers.	Healthcare personnel's need for education in basic palliative medicine<br><br>Healthcare personnel´s need for information
