# Supplementary material for: The needs of healthcare personnel who provide home-based pediatric palliative care: a mixed method systematic review
Source: BMC Health Serv Res. 2024 Jan 9;24:45. doi: 10.1186/s12913-023-10495-7 (PMC10777650; doi:10.1186/s12913-023-10495-7)
Supplement: Supplementary file 5 — Supplementary Material 5 [file 12913_2023_10495_MOESM5_ESM.pdf]

Additional file 5. Examples of stages of thematic synthesis

Data from included studies	Codes	Descriptive themes	Analytical themes
**Qualitative data**<br>The informants experienced it as easier when a child was referred to home care services during the early palliative phase or with specified care tasks before the palliative phase. It allowed them to build a trusting relationship with the family which helped them to shoulder the burden as the child's illness progressed (22).	The need to establish a relationship to the child and family	The need to build a trusting relationship with the child and family	**Being connected and engaged with the child and family**
**"Qualitized" data**<br>Building a close rapport with the family was seen as important of most general practitioners (25).			
**Qualitative data**<br>Developing a good relationship with the family from the outset was seen as essential in order to fully identify and meet their needs (26).			
**"Qualitized" data**<br>The importance of good communication skills was recognized of many general practitioners: being able to talk openly to families and not avoid difficult conversations required careful thought of what, and how, information was communicated (25).	The need to have communication skills to ensure an explorative conversation	The need for being prepared when caring for a child at home	
**Qualitative data**<br>Nurses had referrals for patients with both cancer and noncancer. Some of the illnesses were unfamiliar to them and they felt challenged when parents wanted information about complications of the medical illness and prognosis (23)	The need to know different diseases and the prognosis		
**"Qualitized" data**<br>For half of the nurses who took part in this study, the additional comments emphasized the remuneration did not adequately reflect the time spent at the patient's home and the lack of a difference between adult and pediatric fee scales (18).	The need to have time	The need to respond to the family`s needs in time	
**Qualitative data**<br>Their busy workload made it difficult for nurses to juggle planned home visits for the day and to deal with pediatric procedural emergencies or calls for visits from parents (23)	The need to respond in time		

Data from included studies	Codes	Descriptive themes	Analytical themes
**Qualitative data**<br>Most nurses acknowledged that telehealth allowed for participant inclusion with a wider support network included for palliative care encounters (34).	Telehealth allowed a wider support network	The need for effective collaboration within and across services	**Being part of a dedicated team**
**"Qualitized" data**<br>… healthcare professionals expressed a need for more clarity about possible tasks, responsibilities and commitment of the paediatric palliative care teams itself … (31).	The need to clarify roles and areas of responsibility		
**"Qualitized" data**<br>The health care professionals` satisfaction with all assessed aspects of care improved significantly after involvement of the pediatric palliative home care team (32).	The need for cooperation and support with a specialised pediatric palliative care team	The need for guidance and support from other healthcare personnel	
**Qualitative data**<br>The already well-established debriefing and counselling routines within the home care services team were conceived as especially important tools when caring for the sick child (22)	The need to work as a team for professional and emotional support		
**Qualitative data**<br>Although nurse managers acknowledged that out of hours care was being provided, no formal structure existed to ensure that the service provision was equitable and sustainable (26).	The need to have a formal structure to provide out of hour care	The need to establish an equitable and sustainable service	**Ensuring the quality of home-based pediatric palliative care service**
**Qualitative data**<br>Resources for providing 24-hour End-of-Live-Care, with the ability to rest and relinquish practical and emotional responsibilities, were limited as very small local teams and caseloads were spread over wide geographical areas (29).	The need for a sustainable team		
**Qualitative data**<br>Nurses identified specific barriers to receiving ideal training: lack of time away from professional or personal responsibilities, difficulties subsidizing training costs, lack of awareness of available training opportunities, geographic distance from training opportunities, lack of easy access to centralized resources, perception that hospice agencies do not value such training, and emotional burnout resulting in staff attrition (27).	The need to facilitate training	The need for continuous professional development in pediatric palliative care	
